# Organic crystalline nanoparticles with a long-lived charge-separated state for efficient photocatalytic hydrogen production

**DOI:** 10.1038/s41557-025-02035-z

**Published:** 2026-01-20

**Authors:** Bin Cai, Andjela Brnovic, Mariia V. Pavliuk, Leif Hammarström, Lars Kloo, Sarah A. Barnett, Haining Tian

**Affiliations:** 1 Department of Chemistry-Ångström Lab., Uppsala University, Uppsala, Sweden; 2 Institute for Energy Research, Jiangsu University, Zhenjiang, China; 3 Applied Physical Chemistry, Department of Chemistry, KTH Royal Institute of Technology, Stockholm, Sweden; 4 Diamond Light Source, Didcot, UK

**Keywords:** Photocatalysis, Electron transfer, Light harvesting, Molecular self-assembly, Excited states

## Abstract

Photocatalysis offers a promising approach for renewable energy conversion and storage, but short lifetimes of charge-separated states in photocatalysts due to charge recombination limit its utility. Here we report an organic molecule with an acceptor–donor–acceptor configuration that can self assemble into highly crystalline nanoparticles. Transient absorption spectroscopy reveals that these crystalline assemblies can induce an ultra-long-lived charge-separated state of up to 1.2 s, attributed to initial symmetry-breaking charge separation, followed by charge hopping across closely packed molecules. These self-assembled nanoparticles have an impressive photocatalytic H_2_ evolution rate of 126 mmol g^−1^ h^−1^ with an external quantum efficiency of 12% at 550 nm under optimized conditions. This system shows a remarkable stability with 220 million turnover numbers (per particle) over the 77 h of operation. These findings suggest that rational design of organic molecules and their aggregates is vital for improving light-induced charge separation and for developing highly efficient, stable and scalable organic photocatalysts.

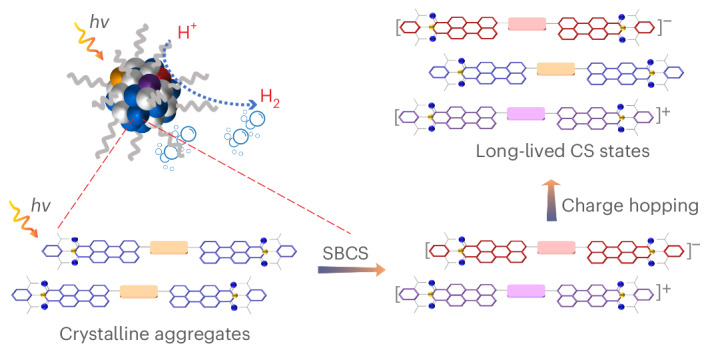

## Abstract (web summary)

Achieving charge separation with minimal energy loss remains a key challenge in photocatalysis, but traditional approaches often suffer from rapid charge recombination or inefficient energy utilization. Now it has been shown that symmetry-breaking charge separation within organic crystalline nanoparticles can generate long-lived charge-separated states, enabling efficient photocatalytic hydrogen production.

## Main

Photocatalysis represents a promising approach for sustainable energy conversion and storage, and organic photocatalysts have great potential for use in photocatalytic applications due to their high extinction coefficients, solution processability and tunable electronic structures^1,2^. However, the weak dielectric screening effect in organic materials often leads to the formation of Frenkel excitons (tightly bounded electron–hole pairs with a binding energy typically around 0.1–1 eV). This leads to ultrafast charge recombination (CR)^3,4^, therefore limiting the photocatalytic performance. Strategies to prevent CR processes primarily include: (1) constructing a donor–acceptor system to accelerate the charge separation^5–8^ and (2) promoting triplet state formation via intersystem crossing, where CR to ground state (GS) is spin forbidden^9,10^. Among these, symmetry-breaking charge separation (SBCS)—in which charge-separated states are formed between identical chromophores via solvation energy or electronic coupling—offers a minimal energy loss pathway, making it particularly attractive for photocatalytic applications^11–13^. However, the related investigations have been either restricted to organic solutions by tuning the solvent polarity or have exhibited a limited photocatalytic performance in aqueous conditions ^14–18^. Developing efficient photocatalytic systems in aqueous conditions is crucial for sustainable artificial photosynthesis, aiming towards using water as an electron and proton donor. Recently, aqueous-phase surfactant-stabilized organic nanoparticles (NPs) have emerged as highly efficient photocatalysts for H_2_ production^19–22^. The small size of these NPs increases their reactive surface area and shortens the charge migration distance. These advantages increase the probability that photogenerated charges can reach the surfaces of the NPs and transfer to the substrate to complete the photocatalytic cycle. Despite substantial progress in the development of various organic NPs for photocatalytic H_2_ production, several critical challenges remain. These include the improvement of photocatalytic performance and enhancing stability, as well as understanding the photophysical properties of organic NPs by structural design and mechanistic studies.

**Fig. 1:**
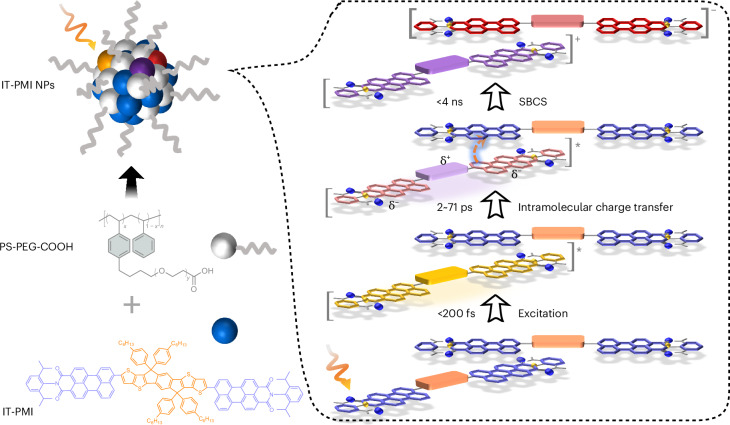
Schematic of IT-PMI NP formation and its photophysical processes. Molecular structure of IT-PMI and schematic representation of photophysical processes within IT-PMI NPs generating a local excited state upon photoexcitation, followed by ICT and SBCS between adjacent molecules.

In this Article, we report the design of an acceptor–donor–acceptor (A–D–A) organic molecule, IT-PMI, which has *C*_2_-axis structural symmetry. IT-PMI comprises an indacenodithieno[3,2-*b*]thiophene (IT) donor core linked to two perylene monoimide (PMI) acceptor arms (Fig. 1). The strong electronic coupling between the donor and acceptor units is expected to promote pronounced intramolecular charge transfer, rendering IT-PMI an integrated chromophore. The extended *π*-conjugation and high structural symmetry facilitate efficient charge transport through strong *π*–*π* interactions and favour the formation of crystalline packing in aqueous media. To realize nanostructured assemblies, IT-PMI was stabilized with an amphiphilic copolymer—polystyrene grafted with polyethylene oxide and carboxyl groups (PS-PEG-COOH)—to form single-component chromophore NPs. These NPs exhibit a nanocrystalline organization consistent with J-aggregate characteristics, where the molecules adopt a slip-stacked, head-to-tail configuration that enables intermolecular excitonic coupling between transition dipoles^23,24^. Such packing is anticipated to give rise to distinctive exciton dynamics, including strong exciton–exciton interactions and singlet–biexciton coherence, markedly different from those in the monomeric state. Through this molecular design and its self assembly, we aim to elucidate the underlying photophysical processes and explore the potential of IT-PMI NPs for photocatalytic H_2_ evolution.

## Results and discussion

### Morphology characterization of the aggregate nanoparticles

**Fig. 2:**
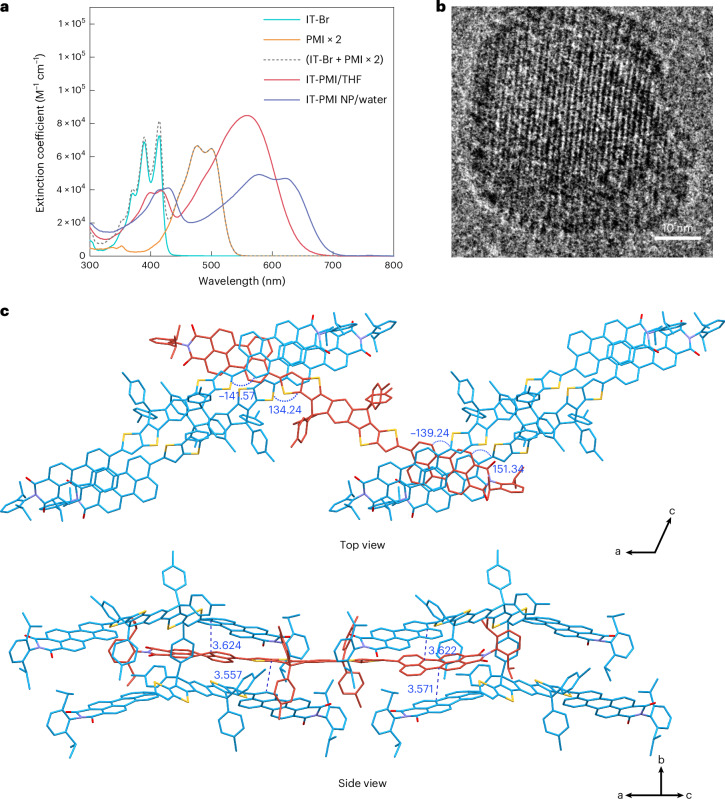
Aggregate packing-style investigation. **a**, UV–vis absorption spectra of the donor core in THF (IT-Br, green line), acceptor arms in THF (PMI, multiplied by 2, yellow line), synthesized IT-PMI in THF (red line) and IT-PMI NPs in water (purple line). **b**, Cryo-EM image of a freeze-dried IT-PMI NP. **c**, Representative molecular packings from single-crystal measurement (alkylphenyl group simplified as methyl). Source data

Figure 1 shows the structures of the IT-PMI molecule and PS-PEG-COOH surfactant as well as a schematic representation of the proposed photophysical processes within IT-PMI NPs. The detailed synthesis of IT-PMI is presented in Supplementary Figs. 1–4. In IT-PMI, the characteristic absorption peaks of the donor core (IT) and the acceptor arms (PMI) at 400 and 500 nm, respectively, became significantly attenuated, and a dominant intramolecular charge transfer (ICT) band emerged at around 550 nm, indicating strong electronic coupling between the electron-donating core and the electron-poor arms^25,26^ (Fig. 2a). The band around 400 nm suggests a perturbed but still predominantly donor-localized transition. Excitation either directly at the ICT band (550 nm), at 400 or at 500 nm, resulted in identical photoluminescence (PL) spectra of IT-PMI. This observation suggests a rapid locally excited (LE) state to ICT state conversion in the IT-PMI monomer. Moreover, the excitation spectra of IT-PMI also align closely with its absorption spectrum across two monitored wavelengths (Supplementary Figs. 5 and 6).

IT-PMI NPs stabilized with an amphiphilic copolymer surfactant PS-PEG-COOH were prepared according to the nanoprecipitation method described in the Supplementary Information. Compared with the IT-PMI monomer in organic solvent, the ultraviolet–visible (UV–vis) absorption spectrum of IT-PMI NPs in water exhibited a new redshifted band at 625 nm, a characteristic behaviour for J-aggregates^27^. The formation of a structure compatible with J-aggregates, rather than the H-aggregates commonly observed in perylene dyes, is likely to be attributed to the increased steric hindrance introduced by the four alkylphenyl substituents on the donor core, a structural feature that has been widely reported to promote J-type packing in similar systems^28,29^. A significant intensity decrease of the ICT band originally present in the IT-PMI monomer was also observed (Fig. 2a). This suggests that the ICT transition in the monomer is now redistributed due to the strong intermolecular coupling from aggregation in the NPs. We compared aggregation characteristics of NPs with the solid-state film obtained by spin coating. A more pronounced aggregation band was observed in the IT-PMI NPs. This probably results from a slower NP self-assembly process during the THF evaporation, which allowed for a more ordered packing process (Supplementary Figs. 7–9). The enhanced aggregation was further confirmed by cryogenic electron microscopy (cryo-EM) analysis of the IT-PMI NPs, where a layer-by-layer molecular packing pattern was clearly observed (Fig. 2b). Statistical analysis of 32 individual nanoparticles showed interlayer distances ranging from 3.5 to 9 Å. In addition, X-ray diffraction (XRD) measurements of the freeze-dried IT-PMI NPs showed strong diffraction peaks, supporting the idea that the NPs have a crystalline-oriented structure. To gain deeper insight into the thermodynamically favoured packing within IT-PMI NPs, we analysed the single-crystal structure. The results show a slipped *π*–*π* stacking arrangement with an interlayer spacing of approximately 3.6 Å. This is primarily governed by cofacial interactions between the perylene moieties in the PMI arms, which exhibit a torsion angle of about 140° (Supplementary Figs. 10–14). This herringbone-like packing corresponds to a slipped J-aggregate configuration, consistent with the redshifted absorption features observed in the aggregated nanoparticles. Moreover, based on the observed packing behaviours, intermolecular electronic couplings for four representative dimer configurations were estimated using density functional theory (DFT) calculations (Supplementary Figs. 15–17 and Supplementary Tables 1–4). The Coulombic couplings were found to be relatively weak, suggesting that energy transfer via dipole–dipole interactions is inefficient. By contrast, the significantly stronger charge-transfer couplings indicate that the dimers are primarily governed by charge-transfer mechanisms. The normalized PL spectra of IT-PMI NPs, when excited at either the donor–core (415 nm) or monomer’s ICT band (570 nm), overlap well with the spectra obtained from exciting the newly formed aggregation band (630 nm) in the NPs. Moreover, the excitation spectra of IT-PMI NPs monitored at two wavelengths (660 and 760 nm) match its absorption spectrum well. These results indicate that all PL emissions originate from the newly formed aggregation state in the NPs^30^. Measurement of the ζ-potential showed that the IT-PMI NPs possess an average ζ-potential of around −25 mV, indicative of good colloidal stability. The IT-PMI films exhibit high hole (7.4 × 10^−5^ cm^2^ V^−1^ s^−1^) and electron mobilities (1.7 × 10^−4^ cm^2^ V^−1^ s^−1^, a higher annealing temperature reached 2.1 × 10^−3^ cm^2^ V^−1^ s^−1^), as determined by space-charge limited current measurements^31^ (Supplementary Figs. 18–21). Given a stronger *π*–*π* stacking in the NPs, one can expect that IT-PMI NPs possess even higher charge mobility compared with that of film.

### Photophysical dynamics in nanoparticles

**Fig. 3:**
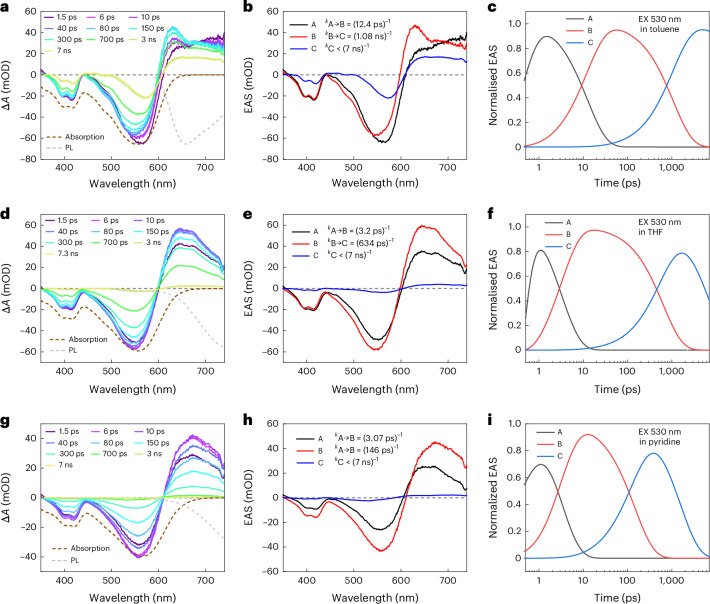
Photophysical kinetics in the monomeric state. **a**,**d**,**g**, fs-TA spectra of IT-PMI measured at an excitation wavelength of 530 nm at ambient conditions and 236 nJ per pulse in toluene (**a**), THF (**d**) and pyridine (**g**). The inverted absorption and PL spectra are shown with dashed lines for comparison. **b**,**c**,**e**,**f**,**h**,**i**, The corresponding global analyses performed with a three-component kinetic model for the evolution-associated spectra (EAS) recorded in toluene (**b**,**c**), THF (**e**,**f**) and pyridine (**h**,**i**). EX, excitation. Source data

To investigate the photophysical properties of the IT-PMI system, steady-state absorption and PL experiments were first performed. While no significant shift in the absorption spectra of the IT-PMI monomer in different polarity solvents was observed, PL spectra exhibited a pronounced bathochromic shift at higher solvent polarity. This behaviour arises because the high structural symmetry of the IT-PMI molecule gives it a weak dipole moment in the ground state, rendering it less sensitive to solvent polarity in the related processes. However, in the excited state, the ICT process induces the formation of quadrupolar or dipolar excited states, which will be stabilized by more polar solvents^32^. Consequently, the Stokes shift increased gradually from approximately 100 nm in toluene to around 250 nm in pyridine (Supplementary Fig. 22). Additionally, both the PL quantum yield and time-correlated single-photon counting (TCSPC) lifetime decreased as solvent polarity increased, suggesting enhanced non-radiative decay via ICT-related pathways^33^ (Supplementary Fig. 23 and Supplementary Table 5). To further investigate excited-state dynamics, femtosecond transient absorption (fs-TA) and nanosecond transient absorption (ns-TA) measurements were performed in three solvents with increasing polarity (toluene, THF and pyridine). The fs-TA spectra and kinetics were independent of excitation wavelength (400 nm at donor–core absorption and 530 nm at ICT absorption), supporting the strong electronic coupling in this A–D–A molecule (Fig. 3). A global analysis of the fs-TA spectra revealed a three-state sequential kinetic model. Upon photoexcitation, state A in all three solvents was characterized by excited-state absorption (ESA) from ~600 nm into the near-infrared (NIR) region corresponding to both LE and ICT states and two ground state bleaches (GSB) at 415 and 560 nm. State A then evolved into a solvent-stabilized ICT state (state B), characterized by essentially the same features as state A, but with intensified ESA and GSB. This conversion was faster in more polar solvents: 12.4 ps, 3.2 ps and 3.1 ps in toluene, THF and pyridine, respectively. In toluene, state B showed a dip in the ESA at 660 nm, arising from stimulated emission (SE) overlapping with the ESA, due to the high quantum yield (high radiative rate constant) in toluene. The stabilized ICT state undergoes CR to a long-lived (>4 ns) state (state C), assigned to a triplet state, probably mediated by spin–orbit charge-transfer-induced intersystem crossing (SOCT-ISC), a widely reported mechanism in donor–acceptor systems^14,16,34,35^. The aforementioned SOCT-ISC accelerates with a more stabilized ICT state in solvents with increasing polarity: 1.1 ns in toluene, 634 ps in THF and 416 ps in pyridine. The previously mentioned triplet state lifetimes were estimated as ~55 μs in toluene, 160 μs in THF and 200 μs in pyridine, monitored at 700 nm using ns-TA spectroscopy. Further evidence for triplet-state formation in the monomer was confirmed by O_2_ quenching experiments, singlet oxygen PL emission in toluene and by a distinct difference of the TA spectra from the sum of difference spectra upon reduction and oxidation in spectroelectrochemistry (SEC) (Supplementary Figs. 24–32).

**Fig. 4:**
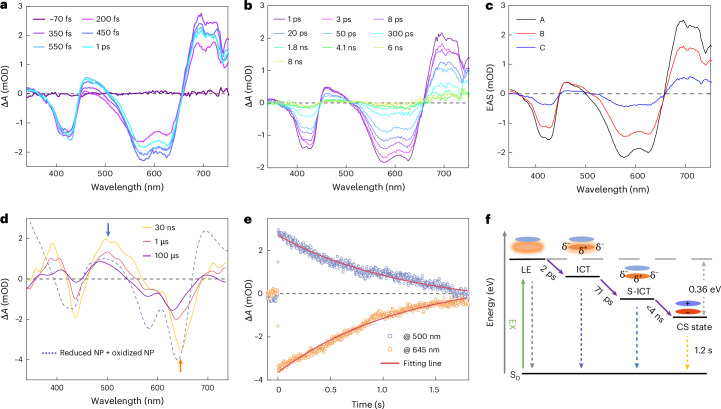
Photophysical kinetics in the nanoparticle state. **a**,**b**, Evolution of fs-TA spectra of IT-PMI NPs in water under ambient conditions during short (**a**) and long (**b**) time ranges, respectively (19.7 nJ per pulse). **c**, Global modelling with evolution-associated spectra. **d**,**e**, ns-TA spectra of IT-PMI NPs in water under Ar conditions (10 mJ per pulse) (**d**) and the selected kinetics traces up to approximately 2 s after ns excitation (**e**). **f**, Scheme of the overall dynamics of IT-PMI NPs, parameters from fs-TA and ns-TA. S_0_, the ground state. Source data

Unlike the monomer, IT-PMI NPs formed well-packed aggregates with strong intermolecular coupling, potentially facilitating electron transfer between molecules within the NPs and exhibiting significantly different excited-state dynamics^36^. The global modelling of fs-TA data for IT-PMI NPs required a three-component model (Fig. 4c). It is important to note that aggregation introduces multiple populations contributing to the signal, reflecting the distribution of states rather than distinct chemical species. Upon 530 nm excitation, state A showed an ESA that extended from 656 nm into the NIR region and GSB peaks at approximately 427 and 577 nm. Moreover, an additional GSB at 624 nm appeared and was assigned to the J-aggregate related state that matched well with the ground state absorption of IT-PMI NPs. Within 2 ps, state B emerged and was characterized by a partial recovery of both GSB and ESA, while the ~470 nm band persisted and redshifted. This suggests that most of the population undergoes ICT within 2 ps, followed by relaxation into a stabilized ICT state (state C) within 71 ps, which exhibited similar spectral features to state B (Fig. 4a,b). Notably, the normalized absorption profile at 4.1 ns diverges substantially from that at 3 ps (ICT state) and closely resembles the sum of the SEC difference spectra of oxidized and reduced IT-PMI NPs (Supplementary Fig. 36). Subsequent ns-TA measurements revealed that the absorption features from ns to μs timescale showed strong agreement with simulated charge-separated (CS) state absorptions obtained from SEC (Fig. 4d). The CS states appear to arise via SBCS followed by charge hopping induced by the close *π*–*π* stacking architecture between molecules within the NPs. Density functional theory analysis indicated that the electronic coupling between adjacent molecules within each dimer layer is dominated by charge-transfer interactions rather than Coulombic coupling. This pronounced charge-transfer character is likely to facilitate the formation of CS states in the aggregates.

To probe the charge recombination dynamics of IT-PMI NPs, we analysed transient absorption (TA) kinetics at 500 nm, attributed to the overlapping signals from oxidized and reduced species and 645 nm, corresponding to GSB, over a timescale of seconds. The decay trace fitted at 500 and 645 nm revealed an ultralong-lived CS state with a lifetime up to 1.2 s (Fig. 4e), compared with CS state lifetimes reported for other organic NPs in water (Supplementary Table 8). The extended lifetime probably results from the charge hopping within the NPs, and induced long distance between charges, therefore reducing the CR rate. To investigate whether a local triplet state is formed in the NPs, a singlet oxygen (^1^O_2_) generation experiment was performed. Notably, no obvious ^1^O_2_ was detected from ^1^O_2_ PL emission in the solution of NPs. However, it could be detected in a toluene solution of the monomer with a quantum yield of around 0.05% at 550 nm. Introducing O_2_ did not cause faster decay of the ns-TA signal. The results imply that there is no or negligible triplet state formation in the NPs. Moreover, continuous-wave electron paramagnetic resonance (CW-EPR) revealed only a symmetric first-derivative Lorentzian signal centred at *g* ≈ 2.00 with a peak-to-peak linewidth of 0.8 mT, characteristic of a stable organic radical^37^ (Supplementary Figs. 29 and 33–35). Based on the Weller equation, the photoinduced charge-transfer driving force in the nanoparticle form of IT-PMI was estimated to be 0.36 eV, significantly higher than that in solution (Supplementary Table 6). A photophysical kinetic scheme of the IT-PMI NPs therefore has been proposed based on the earlier analysis (Fig. 4f).

### Photocatalytic hydrogen evolution

**Fig. 5:**
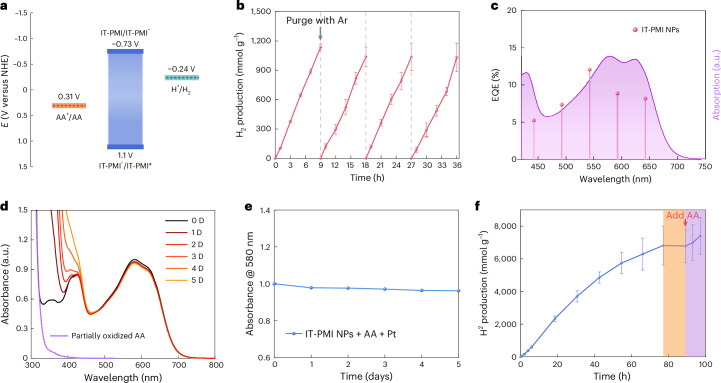
Evaluation of photocatalytic H_2_ production. **a**, Energy level diagram (versus normalized hydrogen electrode, NHE) of the photocatalytic H_2_ production system with AA as the electron sacrificial donor, pH = 4. **b**, Photocatalytic H_2_ production under 50 mW cm^−2^ (420–750 nm) LED light with 45 µg IT-PMI NPs, 6 wt% Pt and 0.25 M AA in 2 ml solution, purging Ar every 9 h to re-initialise the next cycle. Statistics are from 4 independent groups, with a total average HER rate of 112.7 ± 12.3 mmol g^−1^ h^−1^. **c**, External quantum efficiency of H_2_ production based on IT-PMI NPs under optimized conditions, the shadow area is from the absorption profile (with an arbitrary unit, a.u.). **d**,**e**, UV–vis absorbance (**d**) and change traced at 580 nm of IT-PMI NPs dispersed in water with addition of 0.25 M AA and 6 wt% Pt (**e**), illuminated under continuous LED light over 5 days. **f**, Long-term photocatalytic H_2_ production, 2 ml of 23 μg ml^−1^ IT-PMI NPs with 6 wt% Pt, 0.3 M AA, pH = 4. Statistics are from 4 independent groups, with a total average HER rate of 100.1 ± 11.6 mmol g^−1^ h^−1^. Source data

The long-lived CS state encouraged us to further test the system for photocatalytic reaction. The photocatalytic H_2_ evolution reaction (HER) of IT-PMI NPs was then evaluated based on a calibration curve. Energy level alignments were used to determine the thermodynamic driving force for photocatalysis (Fig. 5a) and these are summarized in Supplementary Table 7. Ascorbic acid (AA) served as the sacrificial electron donor, playing a critical role in completing the photocatalytic cycle. AA concentration-dependent HER experiments revealed that H_2_ production follows the Langmuir–Hinshelwood kinetic model as the AA concentration increases (Supplementary Figs. 37 and 38). Consequently, an H_2_ production rate of 126 mmol g^−1^ h^−1^ was achieved using 45 µg IT-PMI in 2 ml aqueous solution with 6 wt% Pt as the co-catalyst. Moreover, after purging Ar through the catalytic system to re-initiate a new cycle, no significant decline in HER performance was observed over three consecutive cycles, indicating the high stability of the prepared IT-PMI NPs (Fig. 5b). The external quantum efficiency (EQE) reached a satisfactory value of up to 12% at 550 nm under the optimized conditions (Fig. 5c).

Cryo-EM images of the Pt-deposited IT-PMI NPs are presented in Supplementary Fig. 39. The H_2_ production rate initially increases with Pt content but decreases once the Pt concentration exceeds an optimal threshold. This decline is likely to be due to either the light-blocking effect caused by surface-covered Pt or an increased charge recombination rate on the Pt surface. Additionally, photocatalytic H_2_ production reaches a plateau when the concentration of IT-PMI NPs exceeds approximately 30 µg ml⁻^1^, which is likely to be limited by light-harvesting efficiency (Supplementary Fig. 40). Importantly, the absence of AA, Pt, light or IT-PMI NPs in control experiments resulted in no detectable H_2_ production, revealing the essential role of these components in the photocatalytic process. We further performed quenching experiments on IT-PMI NPs using ns-TA spectroscopy in the presence of AA or Pt in concentrations used in photocatalytic experiments. The addition of AA or Pt resulted in noticeable spectral and kinetic changes at 500 nm ESA, while there was no obvious change in fs-TA spectra upon the addition of AA or Pt (Supplementary Figs. 41–43). Upon adding AA, the initial spectrum at 30 ns closely resembles that of the absorption of reduced IT-PMI NPs, whereas with Pt, it more closely matches that of the absorption of oxidized IT-PMI NPs.

The stability of IT-PMI NPs is a crucial factor for their scalability in photocatalytic H_2_ production. To assess this, we monitored the absorbance evolution of IT-PMI NPs in Ar-purged water over 5 days, both in the presence and absence of AA and Pt (Fig. 5d,e). In the absence of AA and Pt, the main absorption peak of IT-PMI NPs at 580 nm decreased to 20% of its original intensity after 5 days of light-emitting diode (LED) illumination (Supplementary Figs. 44 and 45). By contrast, with AA and Pt present, the main absorption peak retained over 95% of its initial intensity under identical conditions due to efficient charge extraction from the NPs, avoiding material damage by accumulated charges. Notably, the IT-PMI monomer in dimethylformamide (DMF) exhibited the opposite trend, showing no obvious decay in pure DMF but quick degradation in the presence of AA and Pt. These results further support the idea that the CS within IT-PMI NPs takes place between molecules resulting in free charges. In addition, we conducted a long-term photocatalytic H_2_ evolution experiment, where the reaction ceased after 77 h, due to the depletion of AA in the photocatalytic system. Upon addition of fresh AA, the system resumed H_2_ production (Fig. 5f). IT-PMI NPs exhibited a turnover number (TON) of 2.2 × 10^8^ over 77 h that was calculated based on the total number of NPs, giving a turnover frequency (TOF) of approximately 800 s^−1^. This is comparable with that of [FeFe]-hydrogenase with excess reductant^38^. Our TONs are limited by the rate of light absorption, and may increase further with more intense light if following a linear relationship. Furthermore, we scaled up the photocatalytic H_2_ production using 1.2 mg IT-PMI compound in NPs in 70 ml water, collecting 20 ml of H_2_ gas via the water displacement method in a cylinder after 11 h of illumination (Supplementary Video 1 and Supplementary Fig. 46). These results highlight the scalability and stability of the IT-PMI NP system for photocatalytic H_2_ production.

## Conclusion

In conclusion, we have developed an organic molecule, IT-PMI, featuring an ‘A–D–A’ architecture with *C*_2_ symmetry. Photophysical characterizations indicated that the IT-PMI molecule initially forms LE and intramolecular charge transfer states, which evolve into a solvent-stabilized ICT state with more pronounced charge-transfer character, ultimately yielding a triplet state via the SOCT-ISC mechanism. Upon self assembly into NPs, ordered molecular packing induces intermolecular charge transfer followed by LE and ICT transitions, further stabilized through structural reorganization and charge delocalization. Notably, an ultra-long-lived CS state of up to 1.2 s is observed, presumably arising from charge hopping through the strong *π*–*π* interactions. When employed as the photocatalyst (with Pt as a co-catalyst) for H_2_ production, IT-PMI NPs demonstrate a hydrogen evolution reactivity of 126 mmol g^−1^ h^−1^ and a remarkable EQE of ~12%. A turnover number of 2.2 × 10^8^ per nanoparticle over 77 h confirms their long-term stability and satisfactory reactivity. Further evidence for scalable applicability was provided by the collection of ~20 ml H_2_ gas during an 11 h photocatalytic reaction from only 1.2 mg IT-PMI. Overall, our work elucidates the impact of molecular aggregation on photophysical properties and highlights the potential of rigid *π*-conjugated aggregates for highly efficient photocatalysis. This offers valuable insights for the rational design of advanced organic photocatalysts, with broad implications for photochemical conversion technologies, such as CO_2_ reduction and photodegradation of pollutants.

## Methods

### Preparation of organic crystalline nanoparticles

First, 5 ml of 200 μg ml^−1^ IT-PMI/THF solutionwas mixed with 1 ml of 2 mg ml^−1^ PS-PEG-COOH/THF solution by sonication for 5 min. Then, the mixture was poured into 20 ml distilled water rapidly under sonication and THF was evaporated slowly under an 80 °C water bath for several hours. The obtained nanoparticle aqueous dispersion was filtered with 0.22 μm poly(vinylidenefluoride) (PVDF) membrane and its concentration was estimated with a calibration curve using UV–vis absorption.

### Single-crystal structure analysis

Single-crystal diffraction data for IT-PMI were collected in Experiments Hutch 1 (EH1) of Beamline I19, at the Diamond Light Source using an FFD dual air-bearing, fixed-Χ diffractometer fitted with a Dectris Pilatus 2 M pixel-array photon-counting detector^39^. Data were collected using a wavelength of *λ* = 0.6889 Å, under ambient pressure and at 100(2) K, with the sample cooled using a cryostream^40^. The complete dataset was collected using a series of *φ* and *ω* scans, all with a step size of 0.2° and exposure time of 0.5 s. The collected frames were integrated using XIA2 (refs. ^41,42^) and the data were corrected for absorption effects using an empirical method incorporated within DIALS^43^. The structure was solved by dual-space methods and refined by least-squares refinement on all unique measured *F*^2^ values^44^.

### Photocatalytic H_2_ production

First, 1.5 ml IT-PMI nanoparticles in water and 6 wt% Pt (mass ratio to the nanoparticle, in the form of H_2_PtCl_6_ aqueous solution) were added into a 9 ml vial. The mixture was then purged with Ar for 30 min and then 0.5 ml of 2 M ascorbic acid (pH = 4.3, adjusted using 2 M NaOH) was added after purging with Ar for 30 min. The sealed vial was illuminated with an LED light (under 50 mW cm^−2^ LED white light, 420–750 nm). The produced hydrogen was detected using a gas chromatograph (Thermo Scientific, Trace 1300) by injecting 100 μl headspace gas, and the amount was calculated using a calibration plot.

For molecule synthesis and photodynamic experiment details, please refer to Supplementary Information.

## Online content

Any methods, additional references, Nature Portfolio reporting summaries, source data, extended data, supplementary information, acknowledgements, peer review information; details of author contributions and competing interests; and statements of data and code availability are available at 10.1038/s41557-025-02035-z.

## Supplementary information

Supplementary Information Supplementary Figs. 1–46, Tables 1–8 and Discussion.

Supplementary Video 1 Larger-scale photocatalytic hydrogen production with IT-PMI nanoparticles.

Supplementary Data 1 Crystallographic CIF data.

## Source data

Source Data Fig. 2 UV–vis absorption data, uncropped cryo-EM image and IT-PMI molecule packing.

Source Data Fig. 3 fs-TA spectra and fitting data of IT-PMI.

Source Data Fig. 4 fs-TA spectra and fitting data, ns-TA spectra, fitting and spectro-electrochemistry data of IT-PMI NPs in water.

Source Data Fig. 5 Electrochemistry data of the IT-PMI for energy level calculations, four cycles photocatalytic HER performance, data for EQE calculations, UV–vis absorption data for IT-PMI NPs over long-term illumination, characteristic peak absorption decay data for IT-PMI NPs over long-term illumination and long-term photocatalytic HER performance data for IT-PMI NPs.

## Data Availability

The data that support the findings of this study are available in this article and its Supplementary Information. Source data are provided with this paper.
