# Supplementary material for: Organic crystalline nanoparticles with a long-lived charge-separated state for efficient photocatalytic hydrogen production
Source: Nat Chem. 2026 Jan 20;18(4):723–30. doi: 10.1038/s41557-025-02035-z (PMC13061632; doi:10.1038/s41557-025-02035-z)
Supplement: Supplementary file 1 — Supplementary Figs. 1–46, Tables 1–8 and Discussion. [file 41557_2025_2035_MOESM1_ESM.pdf]

nature chemistry

Supplementary information

https://doi.org/10.1038/s41557-025-02035-z

Organic crystalline nanoparticles with a long-lived charge-separated state for efficient photocatalytic hydrogen production

In the format provided by the authors and unedited

Contents

1 Molecule Synthesis 3

2 DFT Computations 6

3 UV-vis absorption and PL emission 8

3.1 IT-PMI monomeric state in toluene 8

3.2 IT-PMI nanoparticle state in water 9

4 Molecular packing investigation in nanoparticles 10

4.1 Cryo-EM images 10

4.2 XRD pattern of the freeze-dried IT-PMI NP 12

4.3 Single crystal structure analysis 13

5 Electronic coupling DFT calculations 15

6 Excitation spectra of IT-PMI NPs 19

7 DLS and zeta potential 20

8 Electron and hole mobility of the IT-PMI film 21

9 Solvent polarity-dependent spectroscopy measurements 22

9.1 Steady state UV-vis absorption, PL emission spectra and TCSPC lifetime 22

9.2 Fs-TA and ns-TA spectra 24

10 EPR 29

11 Electrochemical and Spectro-electrochemistry tests 30

11.1 Photoinduced charge transfer driving force 30

11.2 Spectro-electrochemistry of IT-PMI monomer and NPs 31

11.3 Electrochemistry of the IT-PMI in solution and IT-PMI film in acetonitrile 32

13 $^1O_2$ probing experiments with ABDA 34

13.1 Absorption with ABDA probe 34

13.2 $^1O_2$ PL emission test 35

14 fs-TA profile comparison of IT-PMI NPs 36

15 Examples for the reported charge separated state lifetime for nano-assemblies 37

16 Photocatalytic H_2 evolution 38

16.1 Standard calibration curve for H_2 amount 38

16.2 HER on varied concentration of AA, Pt, and IT-PMI NPs 39

16.3 Cryo-EM images of IT-PMI NPs with Pt as co-catalyst 40

16.4 Concentration-dependent light harvesting efficiency 40

17 ns-TA quenching experiments with ascorbic acid (pH 4) and Pt 41
18 Stability tests 44
18.1 Light stability test of nanoparticles 44
18.2 Light stability test of IT-PMI monomer 45
20 Setup of scalable HER using IT-PMI NPs 46
21 External quantum efficiency (EQE) measurement 47
22 TON and TOF 47
23 Reference 48

1. Molecule Synthesis

IT-Br (IDTTB6-2Br) was bought from Zhi-yan Chemical and used without further purification, PMI was synthesized according to the previous report[1], $Pd(PPh_3)_4$ was freshly prepared, potassium carbonate, dimethylformamide, palladium chloride, triphenylphosphine, hydrazine monohydride bought from Sigma-Aldrich. ^{1}H-NMR was measured with JEOL 400Y.

(1) Tetrakis(triphenylphosphine)palladium(0), $Pd(PPh_3)_4$

In an argon atmosphere, 1.5 g of triphenyl phosphate and 0.2 g of palladium(II) chloride were dissolved in 12 mL of DMSO. The temperature was then raised to 150 °C and maintained for 2 hours to ensure complete dissolution of all compounds. Subsequently, 4 mmol of hydrazine monohydrate was quickly added, and the flask was allowed to cool slowly to room temperature. After bright yellow crystals formed from the solution, the mixture was filtered, and the filter cake was washed with ethanol and ether. The product was then dried under vacuum for 1 hour. This freshly prepared bright yellow $Pd(PPh_3)_4$ will be used directly in the subsequent reaction.

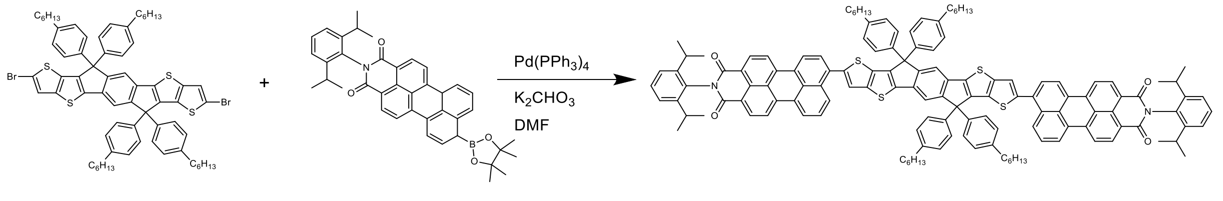

Figure S1. Synthesis route of IT-PMI.

(2) IT-PMI

The synthesis of IT-PMI was conducted following the procedure outlined in Figure S1. 0.93 g PMI and 0.9 g IT-Br were dissolved in 40 mL of N,N-dimethylformamide (DMF) within an argon-purged three-neck flask. The solution underwent argon purging for 30 minutes to remove oxygen. Subsequently, a mixture of K_2CO_3 and $Pd(PPh_3)_4$ was added to the reaction mixture. The temperature was raised to 110 °C and maintained for 6 hours to ensure completion of the reaction. Following the reaction, dichloromethane (DCM) was introduced into the mixture for work-up. The DMF and inorganic salts were removed via washing with water three times. Subsequent to solvent removal using a rotary evaporator, the crude product underwent purification through silica gel chromatography utilizing a DCM:heptane (4:1) eluent mixture. Isolation of the product yielded a dark purple solid weighing 1.17 g, with a satisfactory yield of 77%.^{1}H-NMR (400 MHz, $CDCl_3$) δ 8.67 (dd, J = 7.9, 3.2 Hz, 4H), 8.56 – 8.43 (m, 10H), 7.76

(d, J = 7.8 Hz, 2H), 7.69 (t, J = 8.0 Hz, 2H), 7.58 (d, J = 8.6 Hz, 4H), 7.47 (t, J = 7.8 Hz, 2H), 7.34 (d, J = 7.8 Hz, 4H), 7.27 (d, J = 8.2 Hz, 8H), 7.15 (d, J = 8.2 Hz, 8H), 2.82 – 2.70 (m, 4H), 2.62 – 2.55 (m, 8H), 1.38 – 1.21 (m, 44H), 1.17 (d, J = 6.8 Hz, 24H), 0.86 (dd, J = 9.2, 3.9 Hz, 12H). ^{13}C-NMR (101 MHz, $CDCl_3$) δ 145.776, 142.065, 140.296, 131.105, 128.683, 128.208, 124.110, 35.715, 31.803, 31.385, 29.805, 29.285, 29.228, 24.111, 22.688, 14.196. Chemical formula: $C_{136}H_{124}N_2O_4S_4$, exact mass: 1977.8477, found [M+1]=1978.8528 in high-resolution APCI-MS.

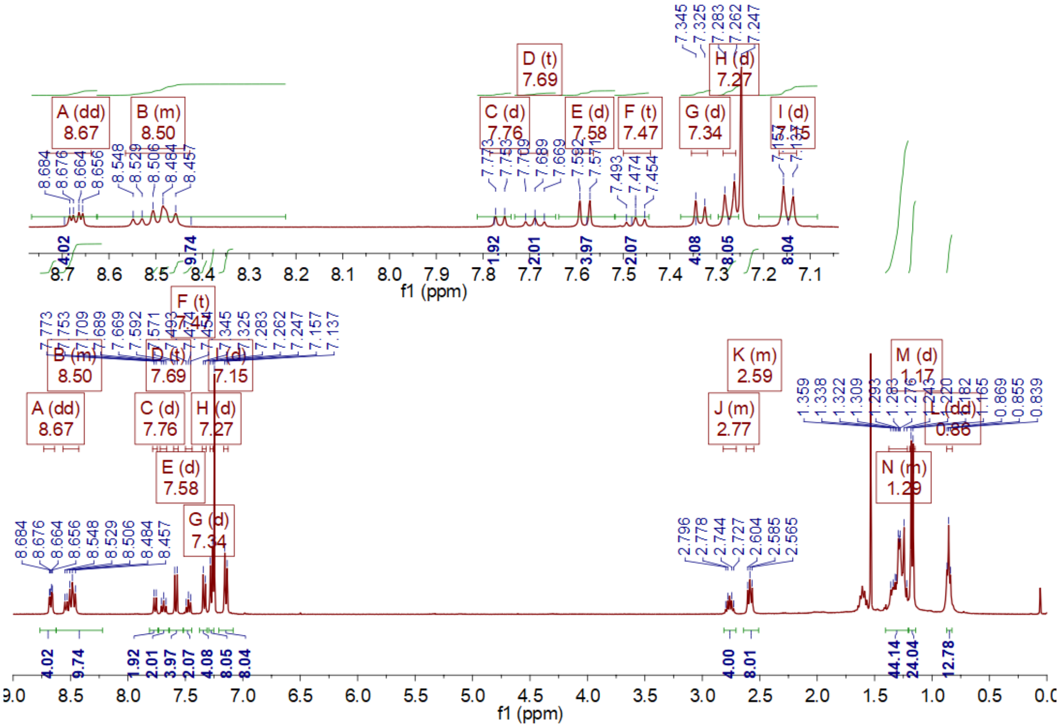

Figure S2. ^{1}H-NMR spectrum of the IT-PMI

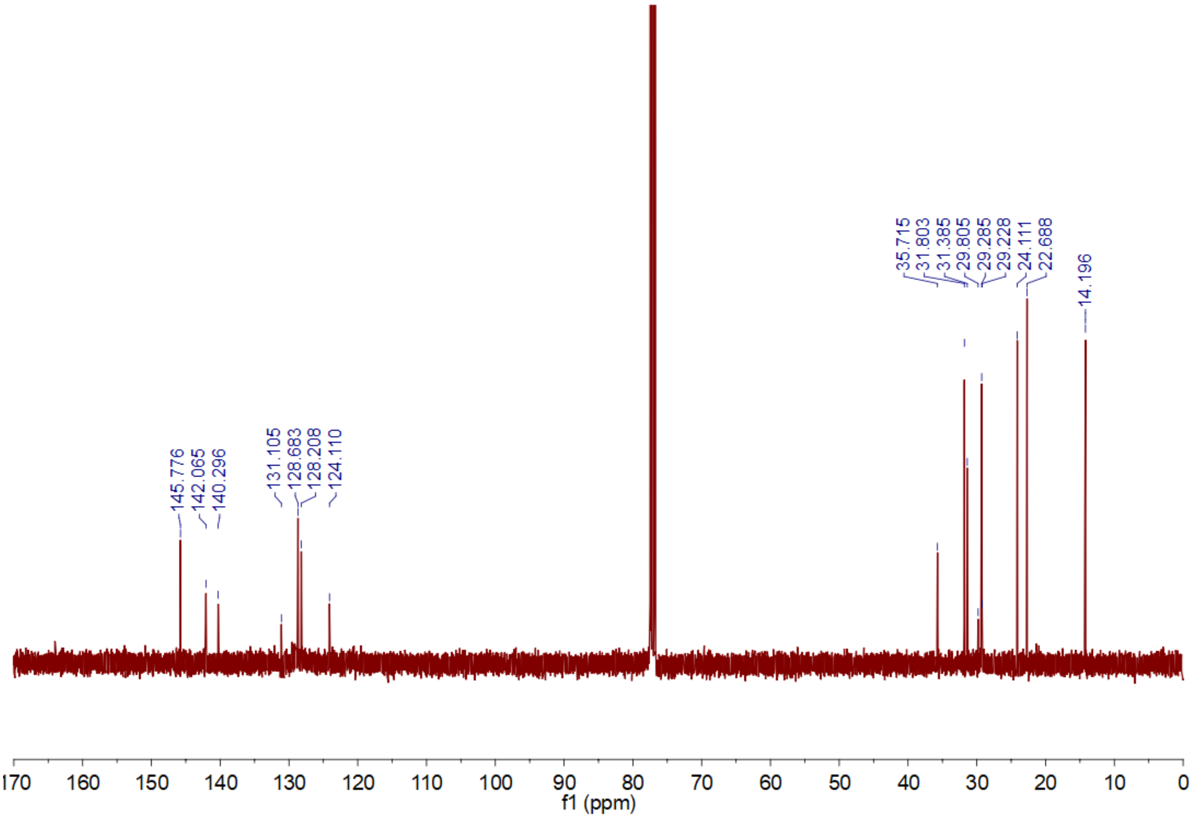

Figure S3. ^{13}C-NMR spectrum of the IT-PMI

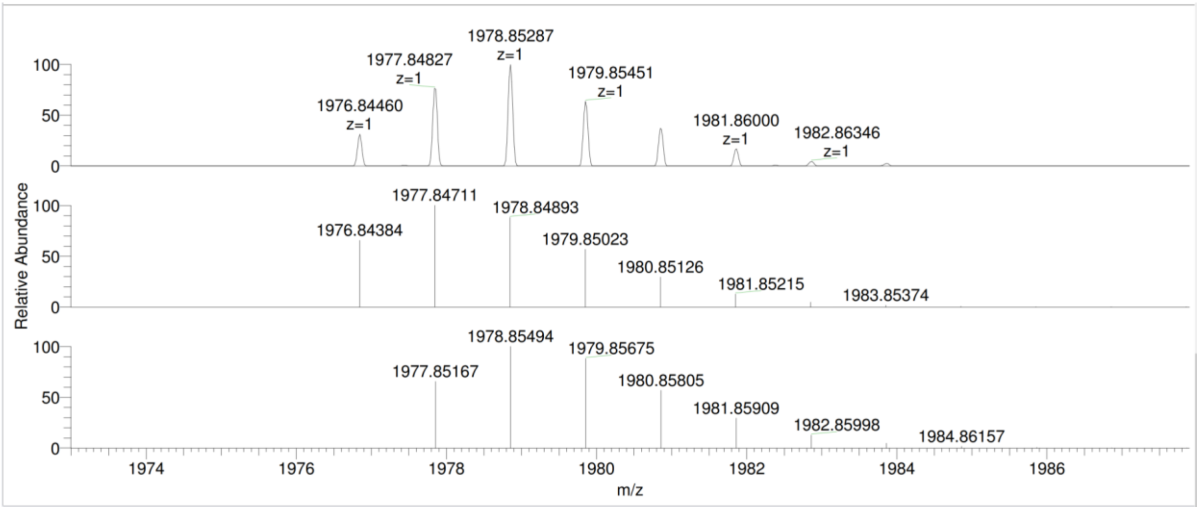

Figure S4. APCI Mass Spectrum of the IT-PMI.

2. DFT Computations

The IT-PMI molecule was modelled using the hybrid density functional cam-B3LYP implemented in Gaussian 16 (Rev. C.01).[2] The molecular structure was geometrically optimized with the simplifications that all hydrocarbon chains were shortened to a methyl group, since these are not expected to significantly change the electronic characteristics of the IT-PMI molecule. Single-point time-dependent calculations (TD-DFT) were performed for the geometrically optimized structures using the cam-B3LYP hybrid functional.[3] 6-311G(d,p) basis sets were used for all the elements (H, C, N, O and S). All results include solvent effects using the dielectric properties of dichloromethane in the polarizable continuum model (PCM).[4] TD-DFT results show that the dominant excited state results from the first singlet→singlet transition ($S_0 \rightarrow S_1$) with an unusually high oscillator strength (f=2.44), indicating a very high molecular extinction coefficient, and the electron transfer from the singlet ground state takes place at 490 nm. The transition is quite complex involving HOMO, HOMO-1 & HOMO-2 → LUMO, LUMO+1 & LUMO+2. The second transition is also a singlet→singlet transition ($S_0 \rightarrow S_2$) corresponding to a 478 nm absorption (f=1.00) being of a similar complexity. The HOMO energy is -6.45 eV, and the LUMO energy is -2.13 eV using the B3LYP functional and including the implicit dichloromethane solvent effects.

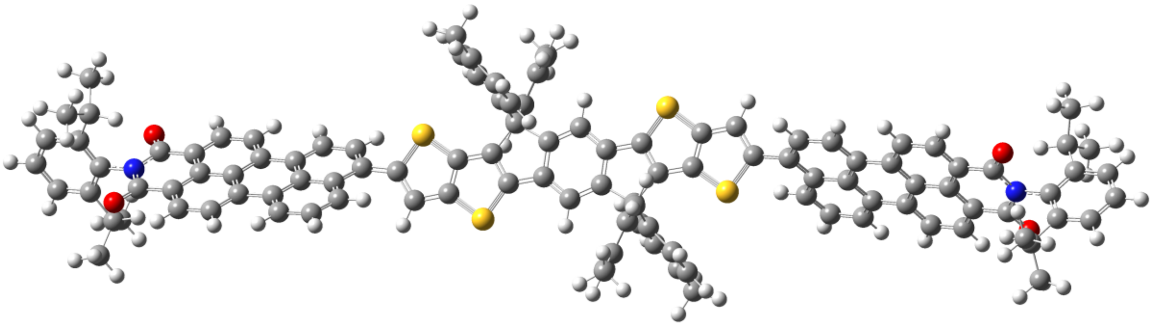

Geometrically optimized IT-PMI structure in DCM solvent (all longer hydrocarbon chains have been replaced with methyl groups).

Table S1. Cam-B3LYP computational results for the electron distribution of the HOMO-2, HOMO-1, HOMO, LUMO, LUMO+1 and LUMO+2 energy level.

LUMO+2	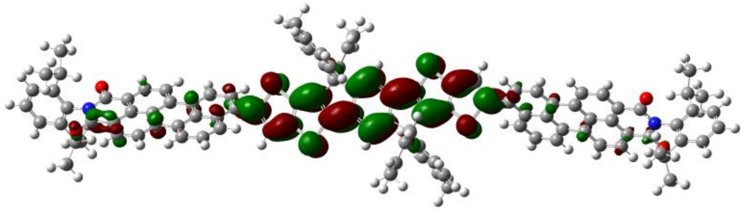
LUMO+1	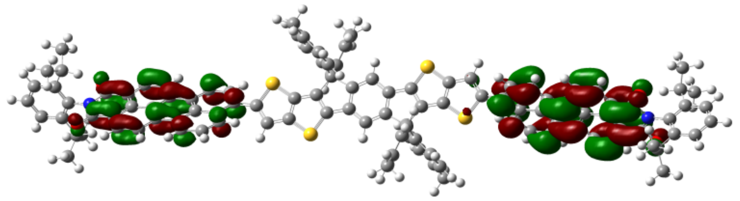
LUMO	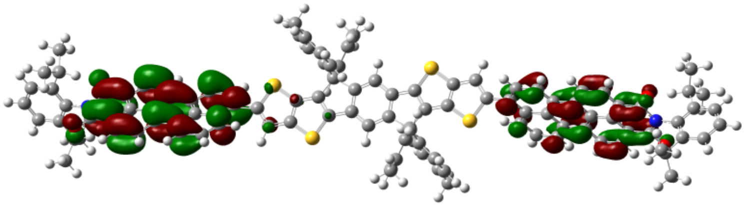
HOMO	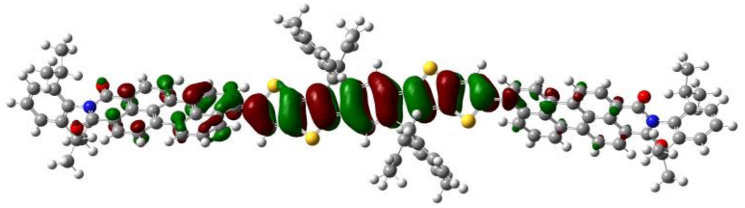
HOMO-1	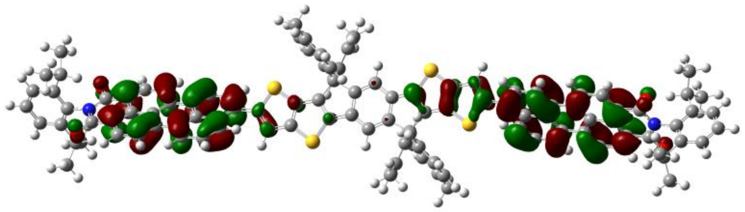
HOMO-2	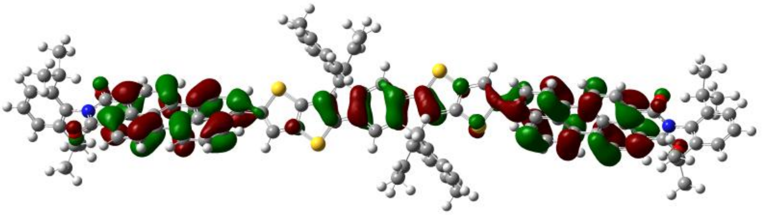
Isolevel 0.02 $[e/(au)^3]^{1/2}$; same isovalue is used in all images	

3. UV-vis absorption, PL emission and excitation spectra

UV-vis absorption spectroscopy was measured with Shimadzu UV-1900i; Steady-state and time-related PL spectroscopy was measured with Edinburgh spectrofluorometer FS5.

3.1 IT-PMI monomeric state

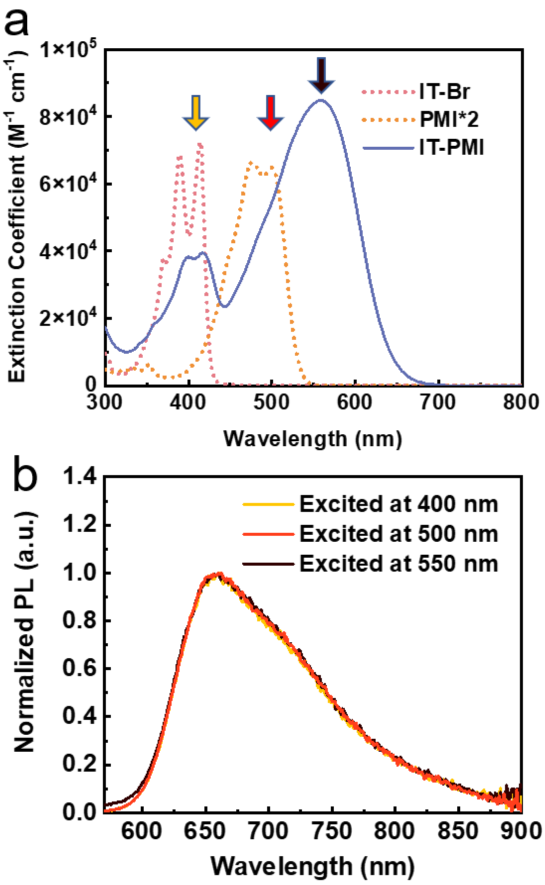

Figure S5. (a) UV-vis absorption spectra of the individual PMI, IT-Br core and IT-PMI measured in Toluene, the extinction coefficient of PMI was multiplied by 2 due to the presence of 2*PMI in IT-PMI; (b) PL emission of IT-PMI in Toluene excited at three selected wavelengths.

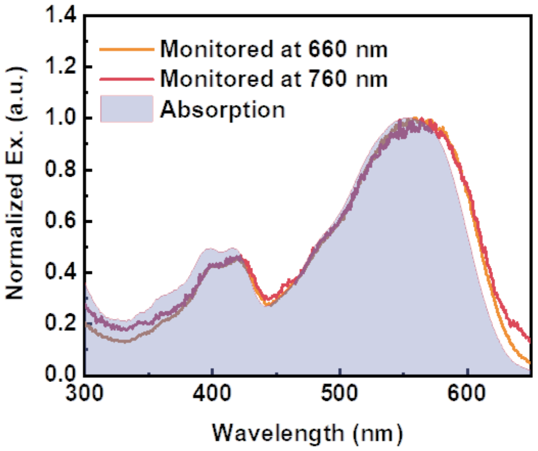

Figure S6. Excitation spectra of IT-PMI in Toluene under two selected monitored wavelengths, the shadow area indicates UV-vis absorption spectrum of IT-PMI in Toluene

3.2 UV-vis absorption spectra comparison of IT-PMI film, IT-PMI NP and IT-PMI solution in Toluene

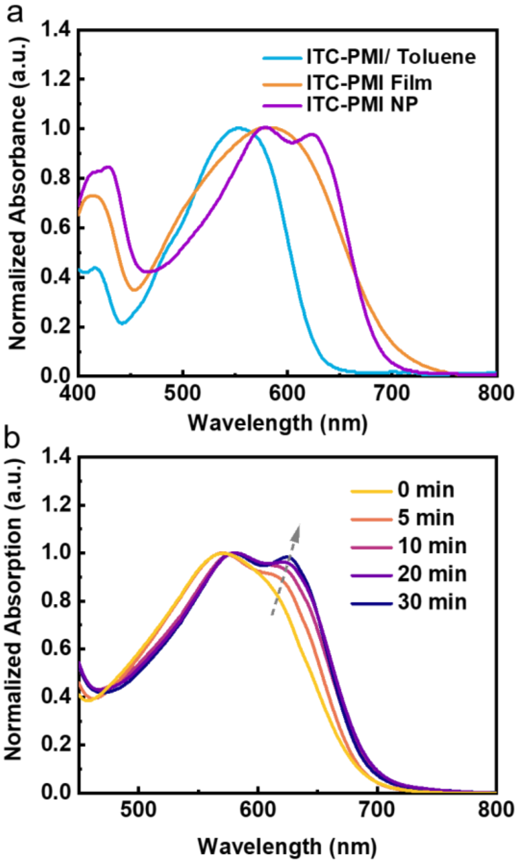

Figure S7. (a) UV-vis absorption spectra comparison of IT-PMI film from spin-coating, IT-PMI NP and IT-PMI solution in Toluene; (b) Enlarged UV-vis absorption spectra evolution of IT-PMI NP in water during THF evaporation process.

Nanoprecipitation method:

5 mL 200 μg mL^{-1} IT-PMI /THF solution was mixed with 1 mL 2 mg mL^{-1} PS-PEG-COOH/THF solution by sonification for 5 minutes. Then, the mixture poured into 20 mL distilled water rapidly under sonification, then THF was evaporated slowly under 80 °C water bath for several hours until no THF was present. After obtaining the nanoparticle water dispersion, the dispersion was filtered with 0.22 μm PVDF membrane.

4. Molecular packing investigation in nanoparticles

4.1 Cryo-EM images

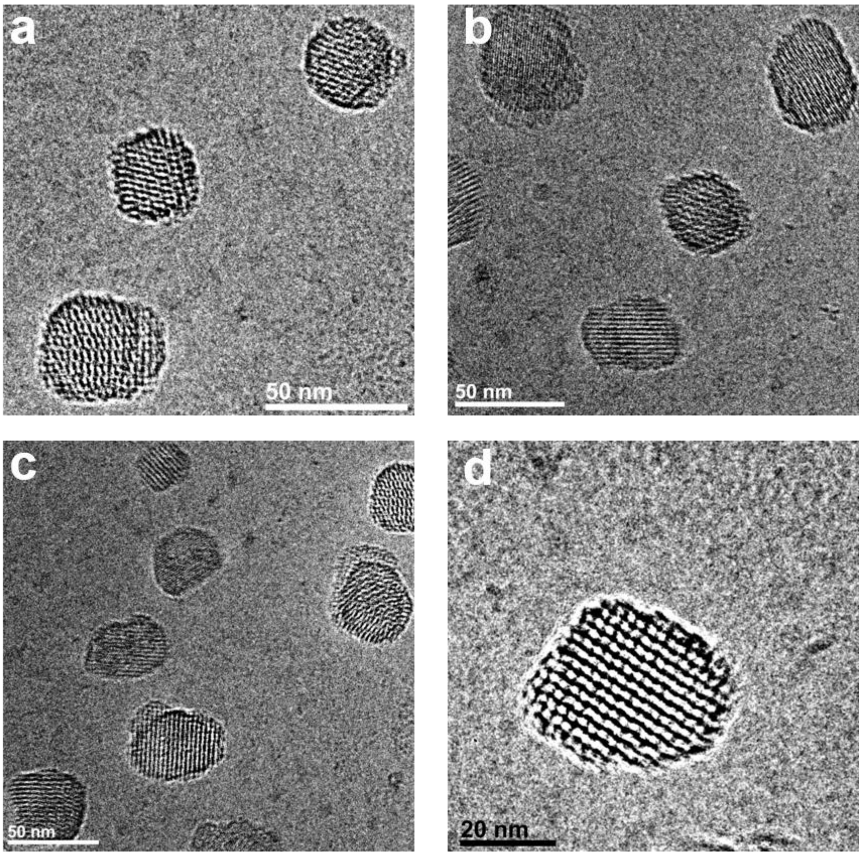

Figure S8. Cryo-EM images of the IT-PMI NPs

Cryo-EM sample was prepared according to previous reports.[5] Briefly, the IT-PMI Pdots were concentrated with centrifuge, to reach a concentration of 1660 μg mL^{-1} (measured with calibration curve). After being frozen, the sample was measured with a Zeiss Libra 120 transmission electron microscope (Carl Zeiss AG, Oberkochen, Germany) operating at 80 kV and in zero-loss bright-field mode. Digital images were recorded under low-dose conditions with a BioVision Pro-SM Slow Scan CCD camera (Proscan Elektronische Systeme GmbH, Scheuring, Germany).

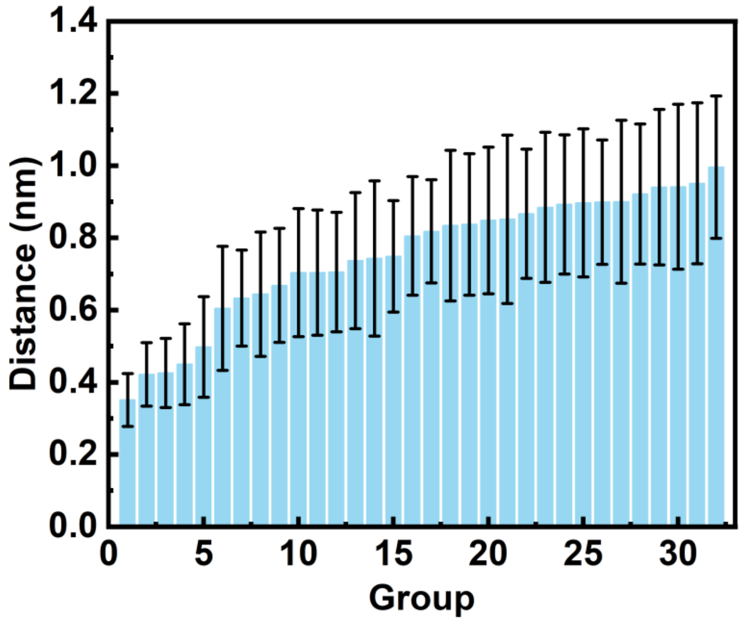

Figure S9. Statistical analysis of the layer-to-layer distances in 32 individual IT-PMI nanoparticles was performed based on cryo-EM images, with each measurement averaged over 50 independent evaluations per nanoparticle.

4.2 X-ray diffraction spectrum of freeze-dried PMI-TP NPs

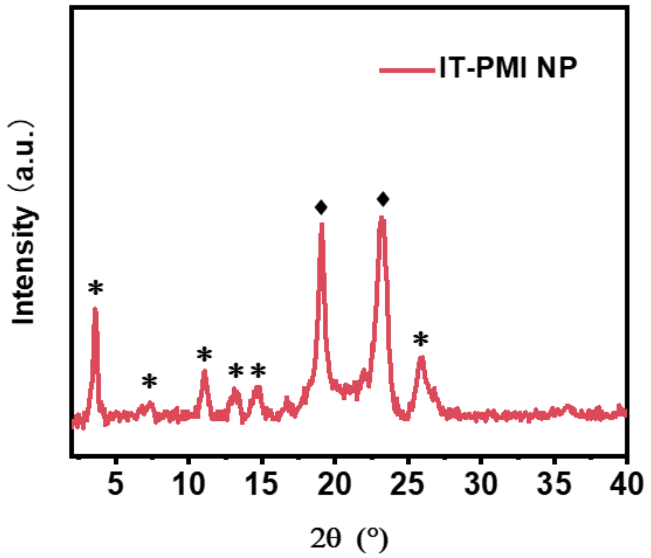

Figure S10. XRD pattern of the freeze-dried IT-PMI NP, * represents IT-PMI NP peak, ♦ represents PS-PEG-COOH peak[6].

4.3 Single crystal structure analysis

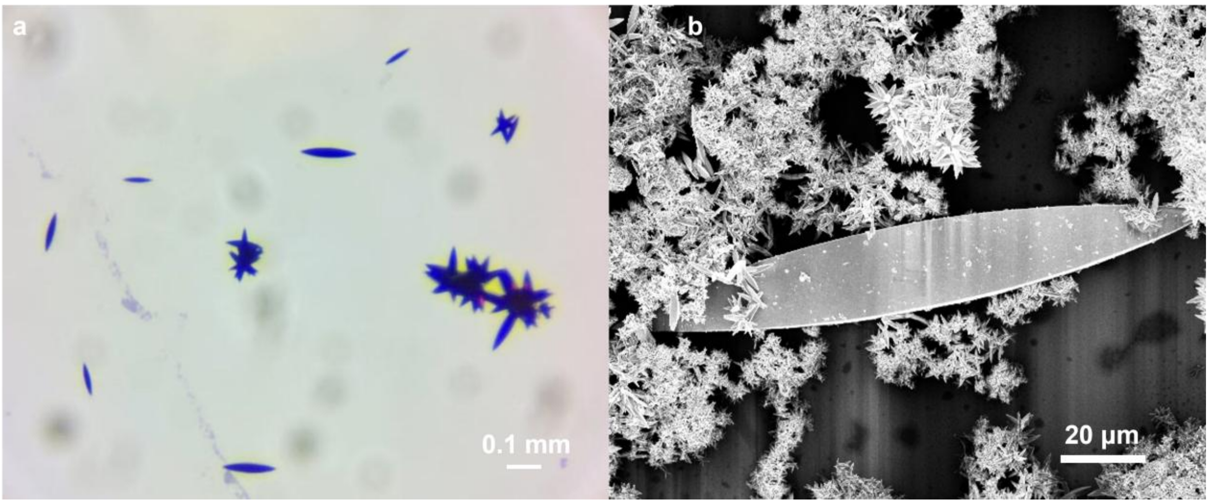

Figure S11. (a) Optical microscope image analysis, scale bar represents 0.1 mm, and (b) Scan electron microscope analysis of the IT-PMI single crystals, scale bar represents 20 μm.

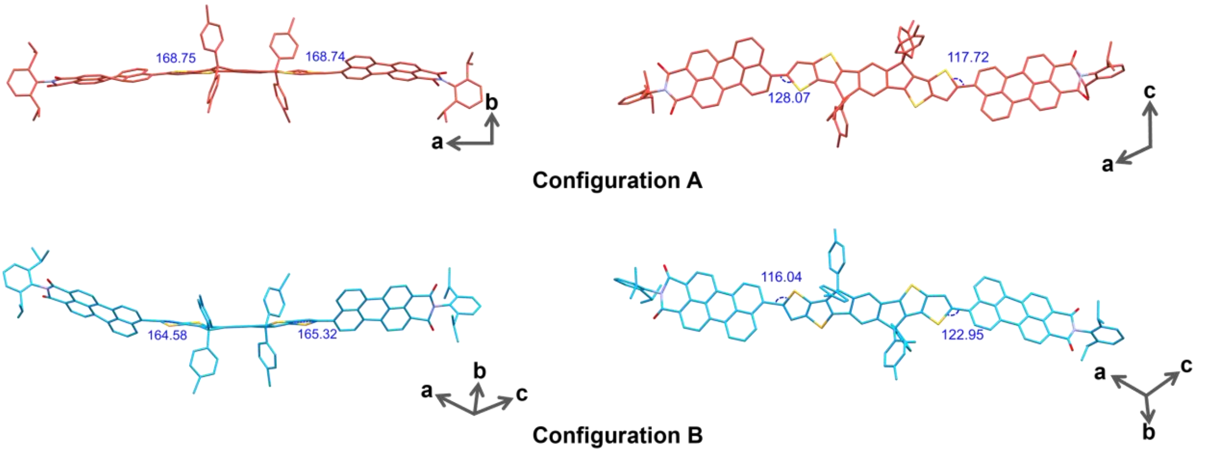

Figure S12. Two different molecular configurations were recognized from the single crystal packing model, obtained from Diamond Light, UK, long alkyl chain and hydrogen atoms are omitted for clarification.

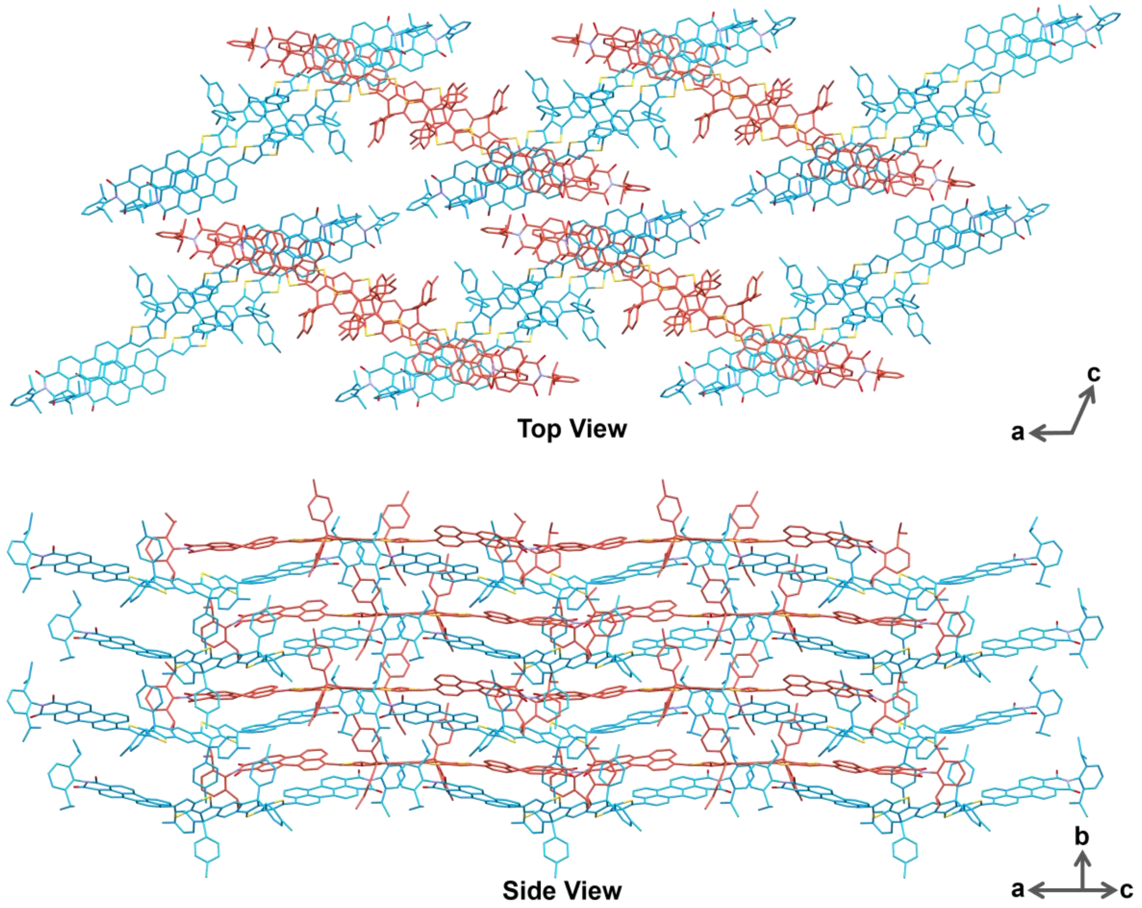

Figure S13. Top view and side view of the molecular packing in the single crystal.

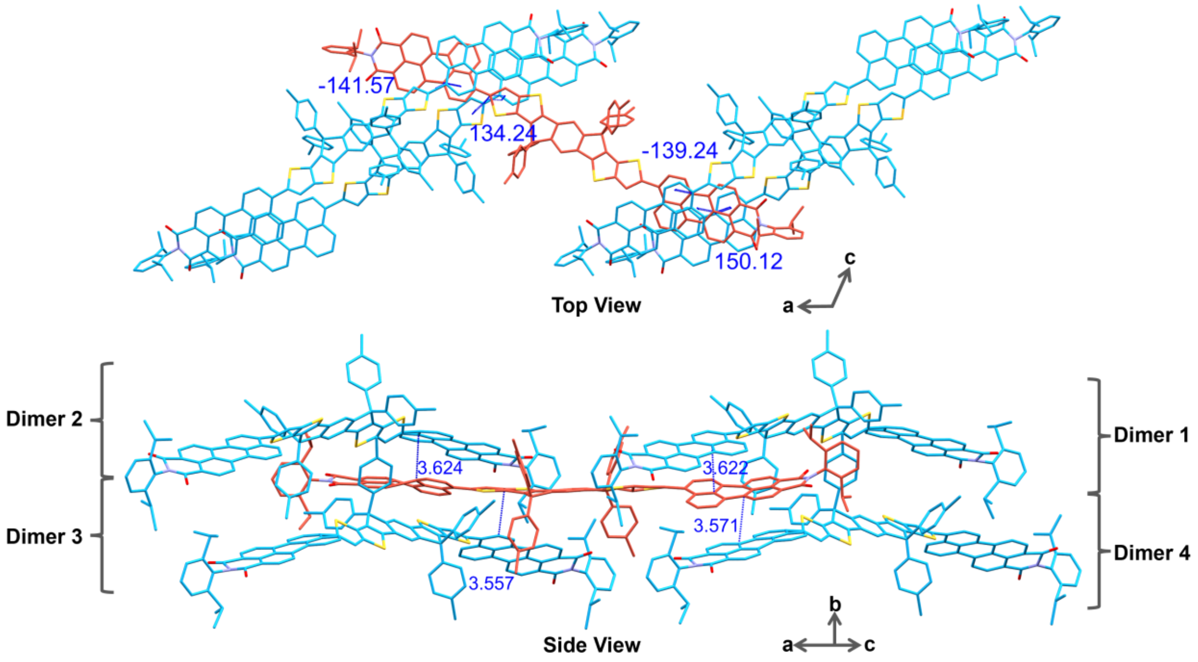

Figure S14. Top and side views of representative dimers, selected based on four distinct intermolecular interaction styles, which served as the basis for the subsequent DFT calculations.

5. Electronic coupling DFT calculations

The charge-transfer and Coulombic couplings were estimated using the same level of computations as noted in HOMO-LUMO calculations. The charge-transfer coupling is quite sensitive to the orbital overlap between the monomers in the four different dimer interaction modes, and thus quite dependent on the distances between the monomeric fragments. It is notable that the closest atom-atom contacts between the monomers are quite short, from H···H contacts at 2.33 Å and H···C/S/O contacts at 2.51 Å and vary in types and distances between the four dimer contact modes as extracted from the crystal structure. The charge-transfer coupling was estimated using the NBO 3.1 routine as implemented in Gaussian 16 based on the fragment orbital approach, where the orbital coupling matrix elements of the two monomers can be identified. The coupling matrix elements can be used to extract the relevant HOMO-LUMO couplings (for electron charge-transfer channels) and HOMO-HOMO couplings (for the hole-transfer channels) between the two fragments/monomers in the four dimer contact modes of the crystal structure and are closely related to the transfer integrals referring to the Marcus theory. The drawback of the use of the natural fragment orbitals is that the exact HOMO and LUMO orbitals cannot be directly identified, and therefore the average of all the relevant matrix elements is given in the table below.

Table S2. Charge-transfer and Coulombic coupling potentials of the four contact dimer modes identified in the crystal structure.

**Interaction**	**Monomer excitations**		**Dimer coupling**		
			Charge-transfer / meV		Coulombic / meV
Blue-Red Dimer	Monomer 1 $S_0 \rightarrow S_1$ / nm*	Monomer 2 $S_0 \rightarrow S_1$ / nm*	holes	electrons	Point dipole
**Dimer 1**	502 (f=3.02)	468 (f=1.66)	290	370	39.3
**Dimer 2**			350	350	45.7
**Dimer 3**			370	360	-91.9
**Dimer 4**			330	390	-19.7

*The two monomer structures are identical in the four different dimer interaction modes generated by the crystal structure packing

The Coulombic coupling, also referred to as electrostatic or Förster coupling, has been evaluated using the results from TD-DFT computations for the $S_0 \rightarrow S_1$ electronic transition based on the monomeric and dimeric structures from the crystal structure packing. Again, the same level of theory was used in these computations, and of course in this case no solvent effects were considered. Coulombic coupling is expected to depend not only on the monomer contact distances, but also on the monomer orientation potentially affecting the direction of the transition dipole moments. The classical point-dipole approximation based on the transition dipole moments of the monomers exploiting the classic Förster equation,[7] and the transition electrostatic potential charges as handled by the Multiwfn 3.8 program.[8] The results are given in Table S 2, and it can be noted that the sign of the coupling potential represents transition dipole directions.

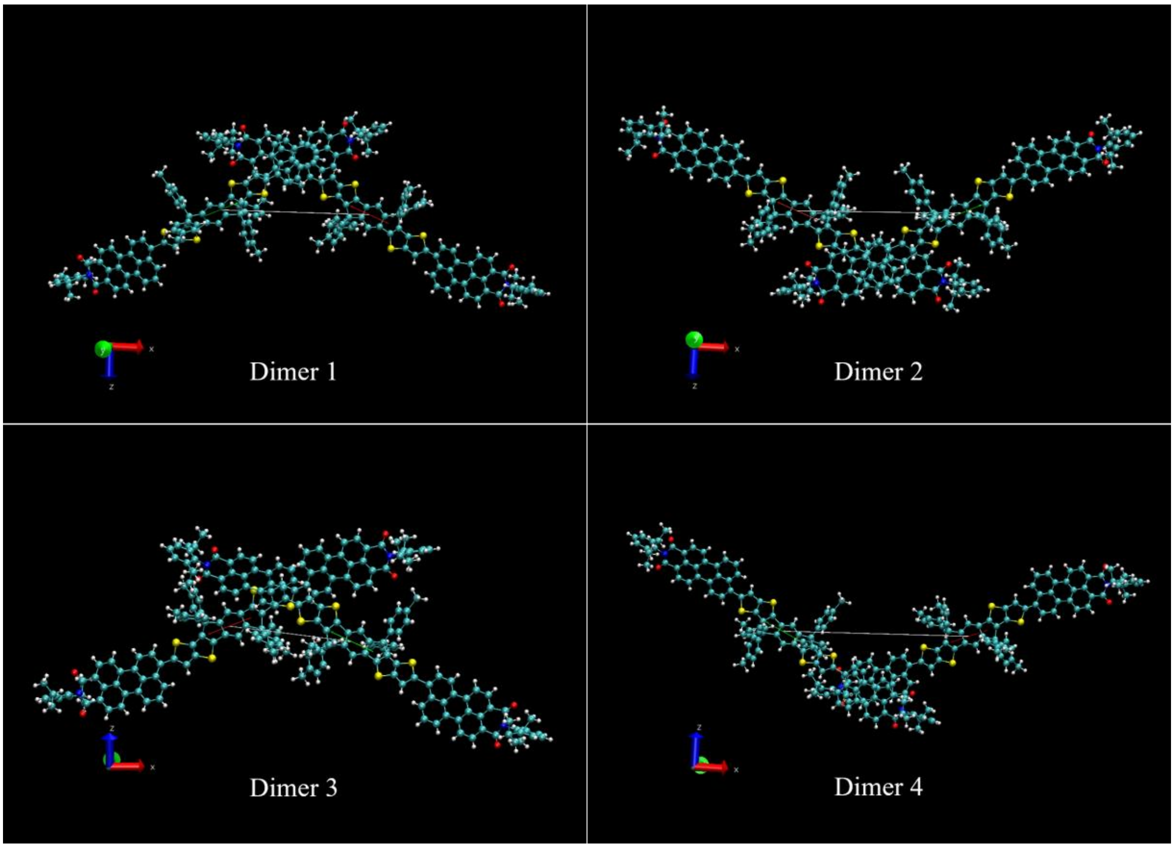

Figure S 15. The transition dipole vectors in 4 dimer monomers from single crystal, quantum chemistry computation from the Förster equation, dipole-displacement vectors (between dipole vector mid-points), red arrow is the transition dipole vector for monomer 1, and the green for monomer 2 (both elongated by a factor of 5 for sake of clarity), and the dipole-dipole mid-point displacement vector in white.

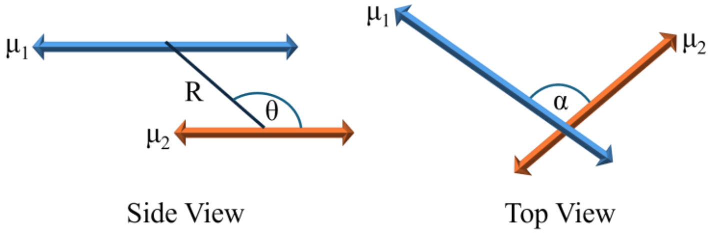

Figure S 16. Diagram of the side and top view of a pair of coupled transition dipole moments in the computed dimers.

Table S 3. Distances, slip angles, and tilt angles between the transition dipole moments of the monomers in the four representative dimers. The coupling constants are negative when the angles exceed 90° and positive when they are less than 90°.

	Distance/ Å	Slip Angle/ °	Tilt Angle/ °
Dimer 1	18.3	24	134
Dimer 2	17.9	157	134
Dimer 3	13.8	148	46
Dimer 4	23.0	21	43

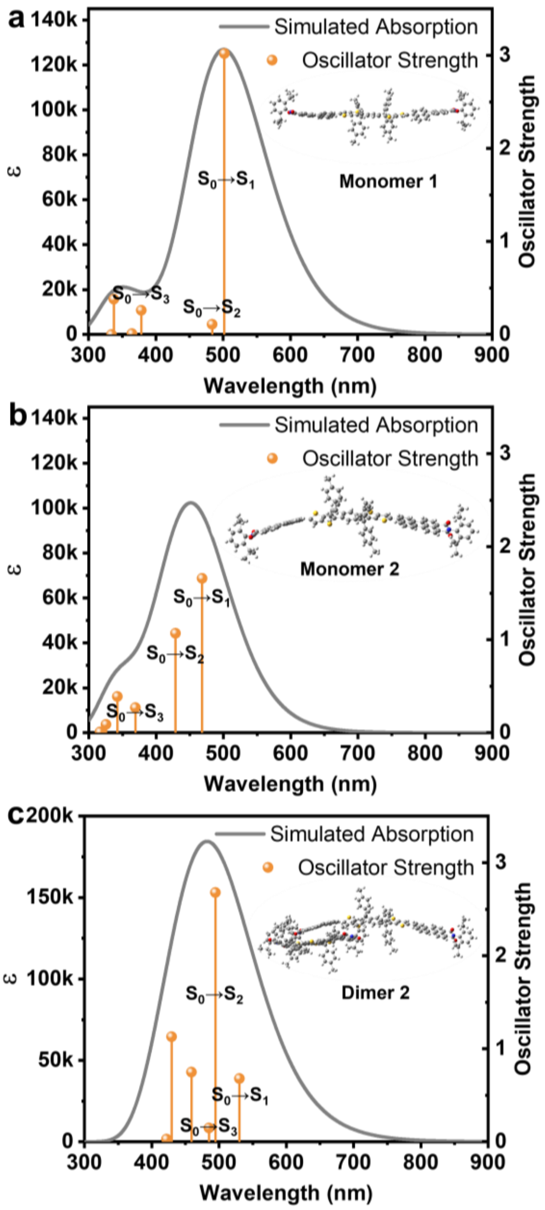

Figure S 17. Computed UV–vis absorption spectra of the representative Dimer2 and its two constituent monomers. Inserted are the molecular structures of Monomer1 and Monomer2, together with their Dimer2 structure determined from the crystal structure. The combined Dimer2 structure shows Monomer2 in essentially the same orientation as in the top, while Monomer1 predominantly points inward.

Table S 4. Summary of the electronic transitions of the three species.

**Transition**	**Monomer1 / nm**	**Monomer2 / nm**	**Dimer2 / nm**
$S_0 \rightarrow S_1$	501.8 (*f*=3.02)	468.3 (*f*=1.66)	530.2 (*f*=0.68)
$S_0 \rightarrow S_2$	483.6 (*f*=0.11)	428.7 (*f*=1.07)	494.5 (*f*=2.68)
$S_0 \rightarrow S_3$	378.3 (*f*=0.26)	369.1 (*f*=0.27)	484.9 (*f*=0.15)
$S_0 \rightarrow S_4$	364.4 (*f*=0.01)	342.3 (*f*=0.39)	458.9 (*f*=0.75)
$S_0 \rightarrow S_5$	337.5 (*f*=0.38)	325.5 (*f*=0.09)	429.4 (*f*=1.13)
$S_0 \rightarrow S_6$	334.0 (*f*=0.00)	316.6 (*f*=0.01)	421.9 (*f*=0.03)

6. PL emission spectra and excitation spectra of IT-PMI NPs at selected wavelengths

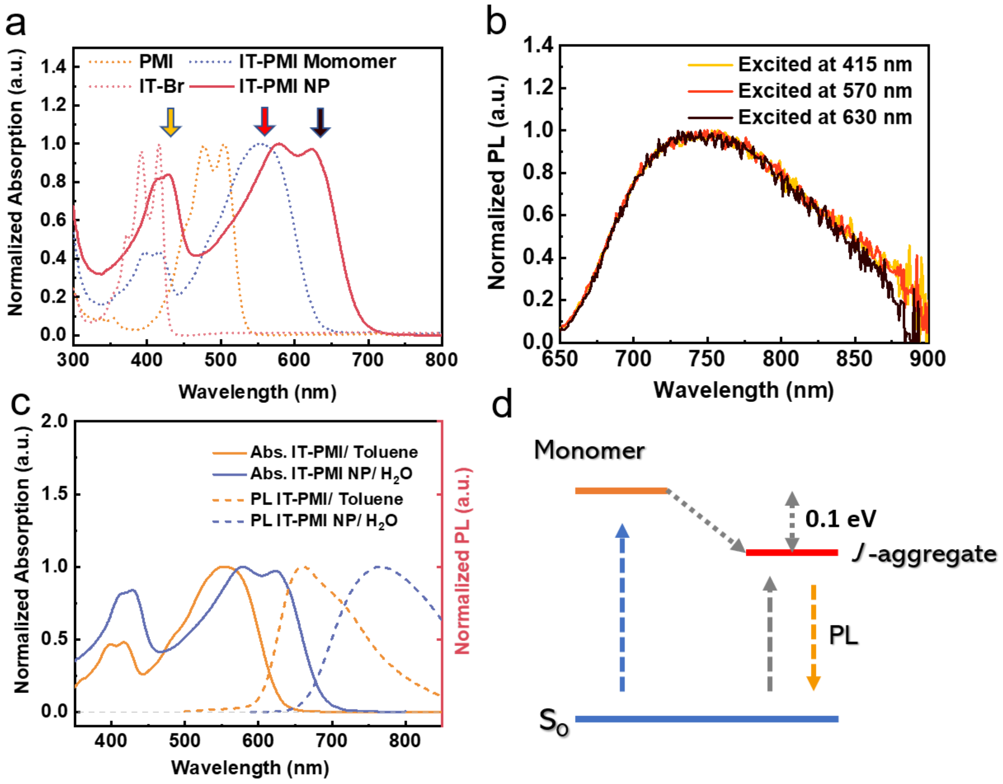

Figure S18. (a) Normalized UV-vis absorption spectra of the IT-PMI NPs, and individual PMI, IT-Br core and IT-PMI measured in Toluene as comparison; (b) Normalized PL emission of IT-PMI NPs in water excited at three selected wavelengths; (c) Normalized UV-vis absorption and PL emission of IT-PMI in Toluene as well as IT-PMI NPs, from which E_{0-0} can be deduced from their cross-section points; (d) Energy level diagram of the proposed IT-PMI monomer and *J*-aggregate.

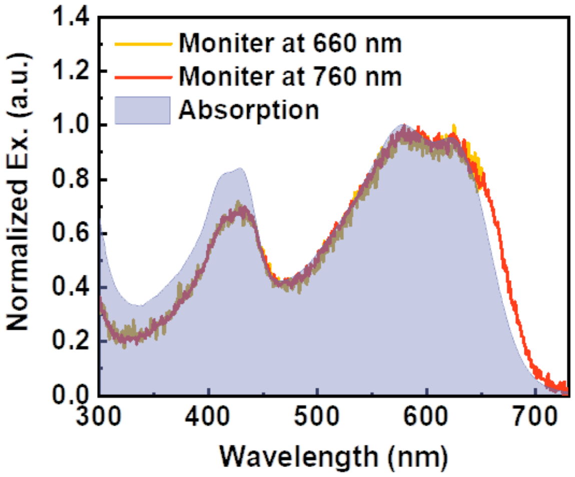

Figure S19. Normalized excitation spectra of IT-PMI NPs in water monitored at two selected wavelengths

7. Dynamic light scattering and zeta potential of the prepared IT-PMI NPs

Dynamic light scattering spectroscopy was measured with Malvern Zetasizer Nano ZS.

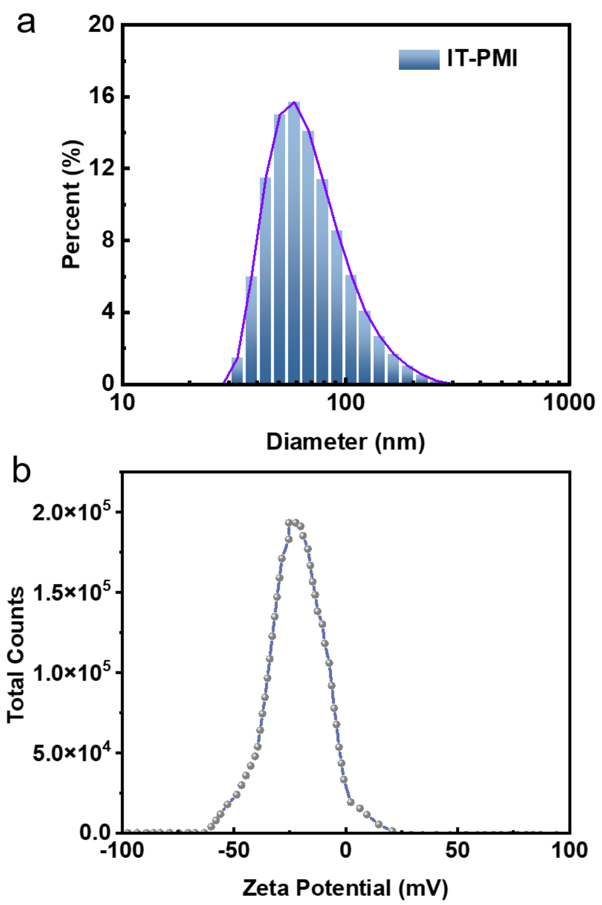

Figure S20. DLS hydrodynamic NP size and zeta potential of the prepared IT-PMI NPs.

8. Electron and hole mobility of the IT-PMI film

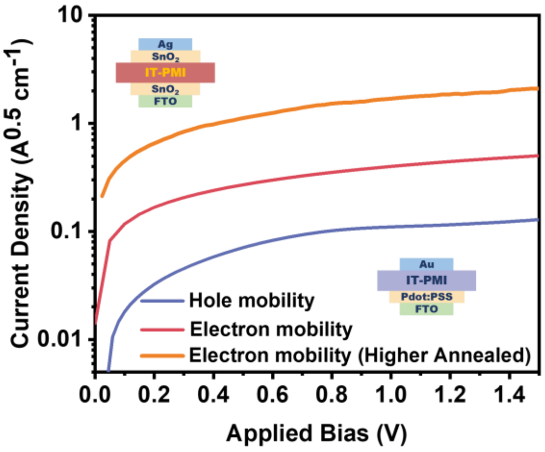

Figure S21. Electron mobility and hole mobility of the IT-PMI film spin-coated on FTO, determined by space-limited charge current measurements.

The mobility value calculated with $J = (9\ \varepsilon_0\ \varepsilon r\ \mu\ V^2) / (8\ L^3)$,

J is the current density (A/m^2)

ε_0 is the permittivity of free space ($\approx 8.854\times10^{-12}$ F/m)

ε_r is the relative permittivity of the material, take 3 for organic semiconductor

μ is the charge carrier mobility (m^2/Vs)

V is the applied voltage (V)

L is the thickness of the material or device (m), 100 nm determined by SEM cross section

9. Solvent polarity-dependent spectroscopy measurements

9.1 Steady state UV-vis absorption, PL emission spectra and TCSPC lifetime

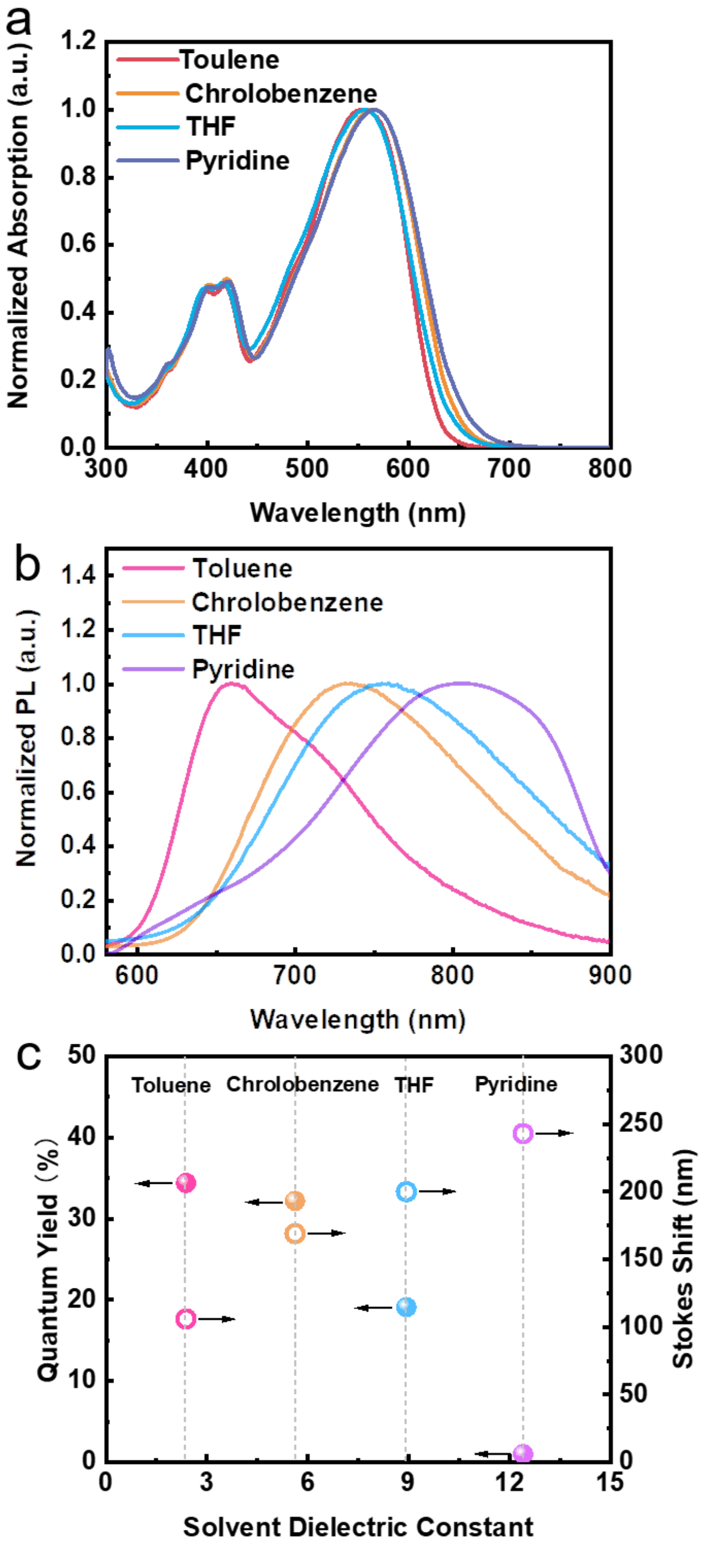

Figure S22. (a) UV-vis absorption spectra of IT-PMI in various polarity solvents; (b) PL emission spectra of IT-PMI in various polarity solvents; (c) PL quantum yield and Stokes shift of IT-PMI in various polarity solvents.

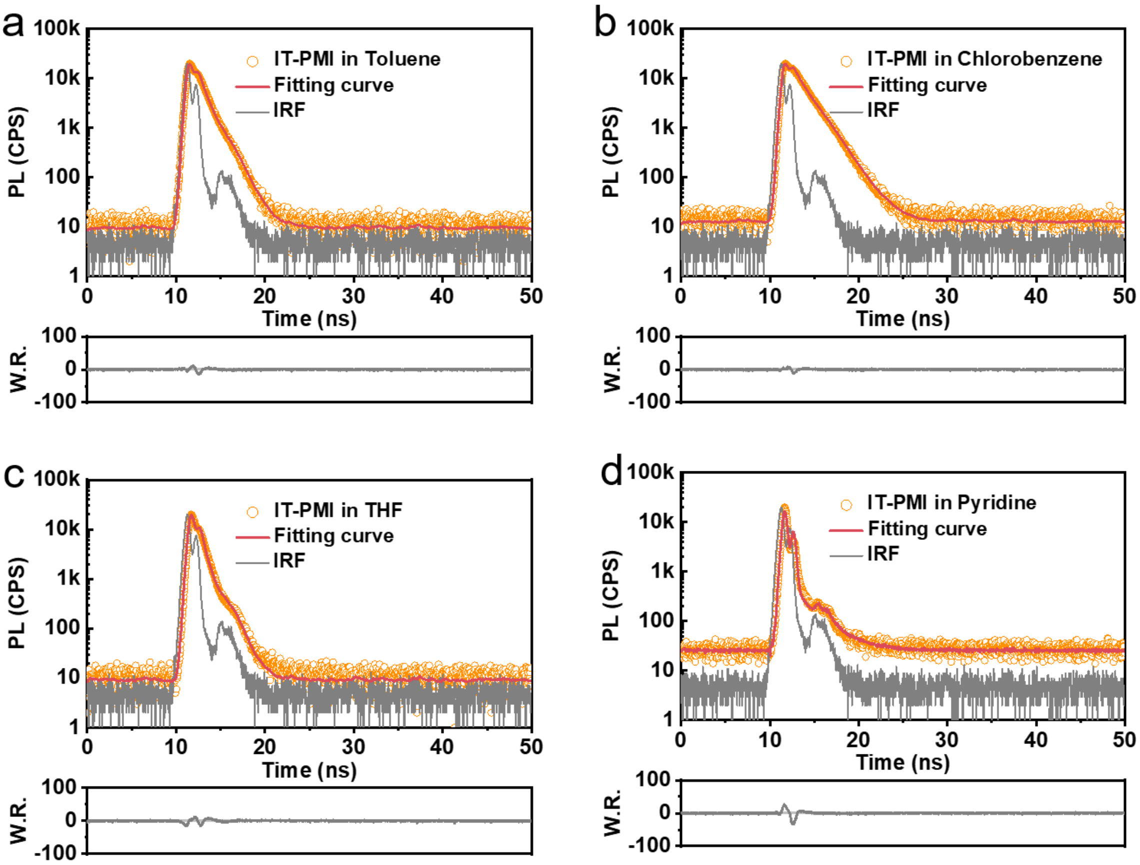

Figure S23. TCSPC measurements of IT-PMI in various polarity solvents.

Table S5. PL related parameters of IT-PMI in various polarity solvents

**Solvent**	ε	QY	$T_{average}$/ ps	K_r+K_{nr}/ s^{-1}	K_r/ s^{-1}	K_{nr}/ s^{-1}	K_{nr}/K_r
**Toluene**	2.38	0.344	660	$1.52*10^9$	$5.23*10^8$	$1*10^9$	1.92
**Chlorobenzene**	5.62	0.322	920	$1.08*10^9$	$3.48*10^8$	$7.3*10^8$	2.09
**THF**	8.93	0.191	410	$2.44*10^9$	$4.66*10^8$	$1.97*10^9$	4.19
**Pyridine**	12.4	< 1%	#	#	#	#	#

9.2. Fs-TA and ns-TA spectra

fs-TA

TA experiments were performed using a Ti:sapphire-based amplifier that included oscillator and pump lasers (Libra, Coherent). The laser's fundamental output (800nm, 3 kHz) was divided into a pump and probe beams using a beam splitter, and these were directed towards a UV−vis−NIR TA spectrometer (TAS, Newport Corp.). Pump wavelengths were obtained through optical parametric amplifiers (400 and 530 nm) (TOPAS NirUVis, Light Conversion). Before reaching the sample, the pump beam passed through a depolarizer and was attenuated with a neutral density filter. The probe supercontinuum was generated from a calcium fluoride crystal (UV−vis), and its path was controlled using an optical delay line with a time window of up to 8 ns. The broadband UV-Vis probe was then focused onto an optical fiber coupled to a MS260i spectrometer (Newport Corp.) after passing through the sample placed in quartz cuvette (1 mm pathlength). The instrument response function (IRF) was 130−150 fs. Sample absorbance was adjusted to approximately ~0.1−0.6 at the excitation wavelength. The measurements were conducted with pump powers varying from approximately 19.7−263 nJ/pulse, focused on the sample in an approximately 0.1 mm^2 spot. The TA data were analyzed using Surface Xplorer software for background removal, time-zero correction, and for fitting the chirp to a third-order polynomial. The scans were carefully reviewed for inconsistencies, with no signs of photodamage. Global analysis was performed by least-squares fitting using the R package TIMP and its GUI Glotaran. A sum of exponentials with wavelength-dependent amplitudes were fitted to the transient data using a parallel scheme, yielding evolution-associated spectra (EAS). All experiments were performed in water, toluene, pyridine and THF at an ambient temperature.

ns-TA

Nanosecond transient absorption (ns-TA) measurements were carried out using the following set-up: Optical excitiation was achieved by a Nd:YAG laser/OPO combination (Ekspla, NT342B laser) that created ≈10 ns pulses at 7.5–10 mJ/pulse at 530 nm. The probe light source consisted of a 450 W ozone-free xenon arc lamp, operated in pulsed mode within an LP920 flash photolysis spectrometer (Edinburgh Instruments). The probe beam was directed perpendicularly to the excitation laser as it passed through the sample. A symmetrical Czerny-Turner monochromator (TMS300) was used to select the detection wavelength, maintaining a spectral bandwidth of 10 nm. Kinetic traces at a single wavelength were recorded using an LP900 photomultiplier detector and subsequently digitized with a Tektronix TDS3012C oscilloscope. For full spectral acquisition, an Andor SH720 ICCD camera was employed. Data collection and processing were performed using the L900 software package. For kinetic traces on second time scales, a Quantel, Brilliant B laser was used. The sample was excited at 532 nm, which corresponds to the second harmonic of the 1064 nm fundamental output. The sample was probed using an unpulsed Xe arc lamp perpendicular to the excitation light. The probe light was passed through two monochromators (Applied Photophysics, pbp Spectra Kinetic Monochromator 05-109) minimize sample excitation by probe light with one before and one after the sample set to 2-mm slit openings. The signal at specified wavelengths was monitored with a photomultiplier tube (PMT, Hamamatsu R928) and digitized using an Agilent Technologies Infinium digital oscilloscope (600 MHZ). Transient absorption data were processed using the Applied Photophysics LKS software. All transient absorption measurements were performed at room temperature using a 1.0 cm path length quartz cuvette. Prior to measurement, all solutions were degassed with Ar.

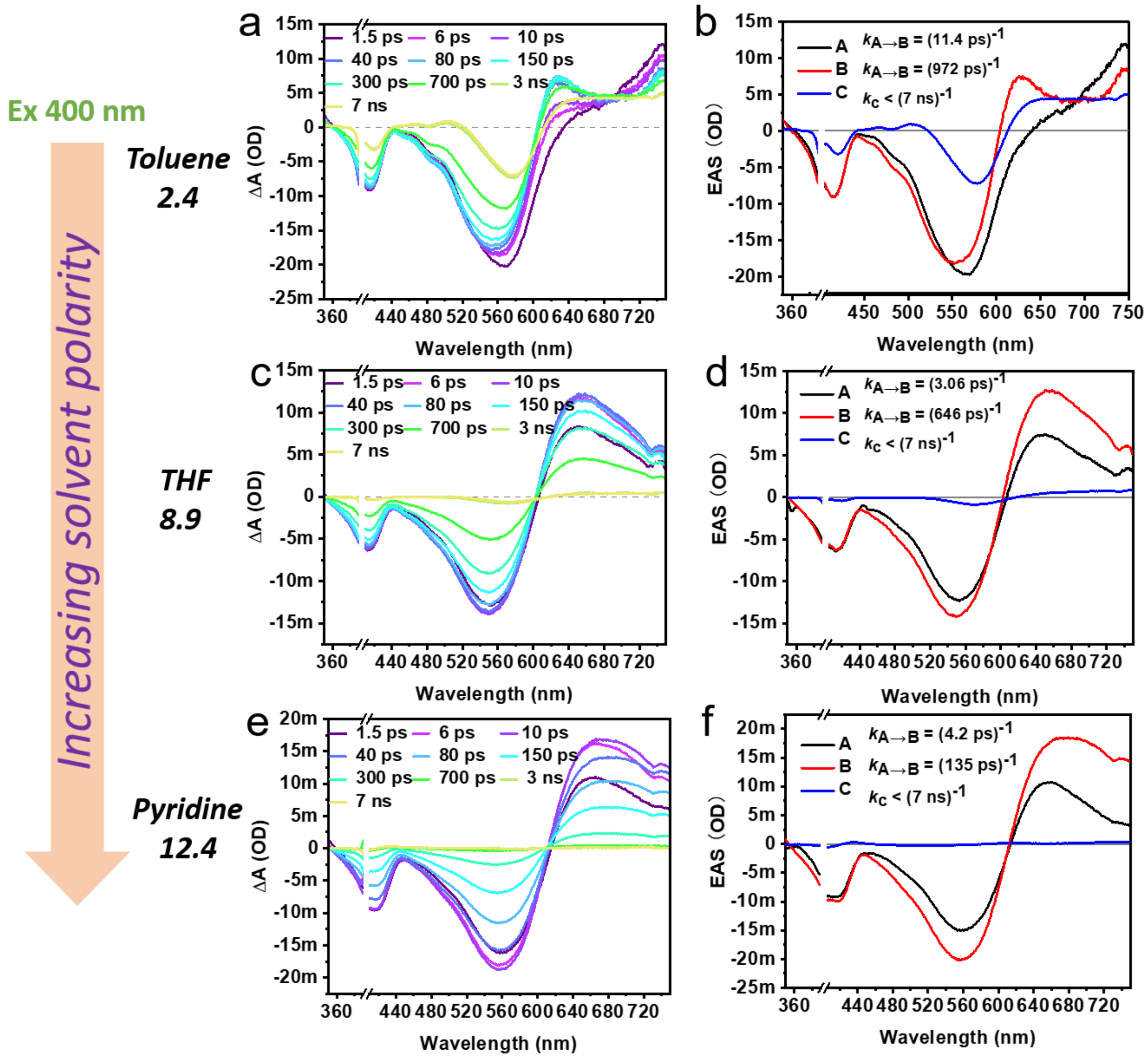

Figure S24. fs-TA spectra of IT-PMI recorded upon 400 nm excitation (263 nJ pulse^{-1}) in (a) toluene, (c) THF, and (e) pyridine. Panels (b), (d), and (f) show the corresponding global analyses performed using a three-component kinetic model for spectra recorded in toluene, THF, and pyridine, respectively.

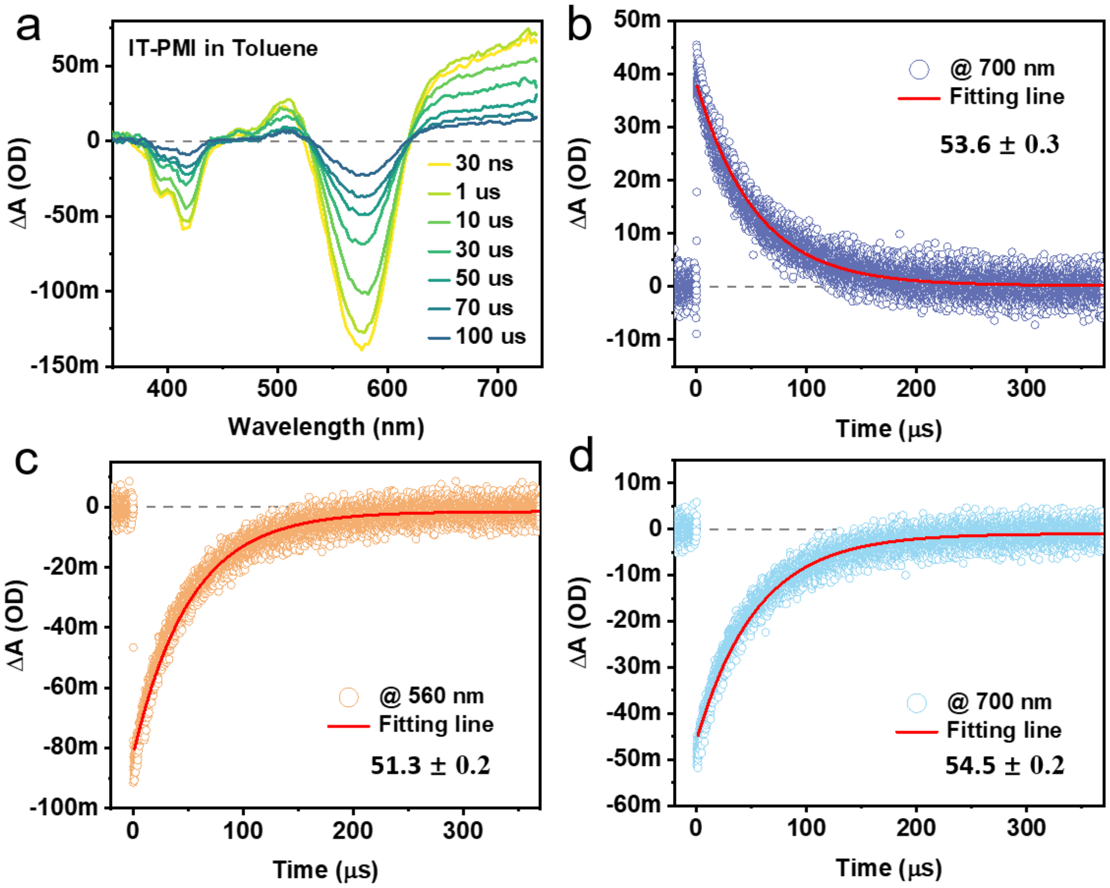

Figure S25. ns-TA spectra and selected kinetics of IT-PMI in toluene, excitation at 500 nm (7 mJ pulse^{-1}).

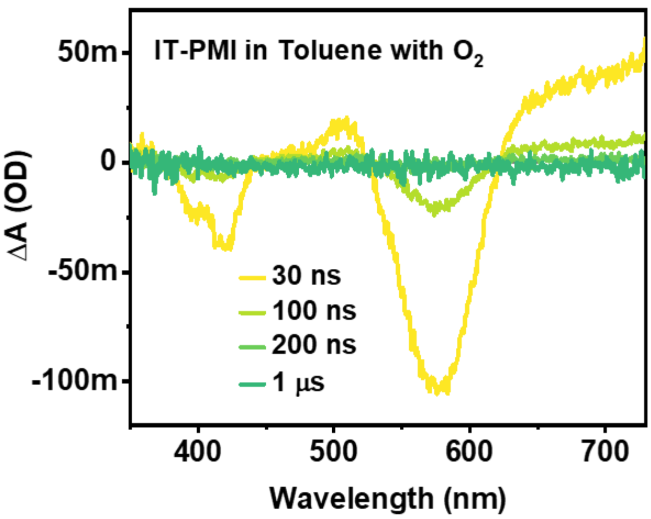

Figure S26. ns-TA spectra of IT-PMI in toluene after O_2 exposure, excitation at 500 nm (7 mJ pulse^{-1}).

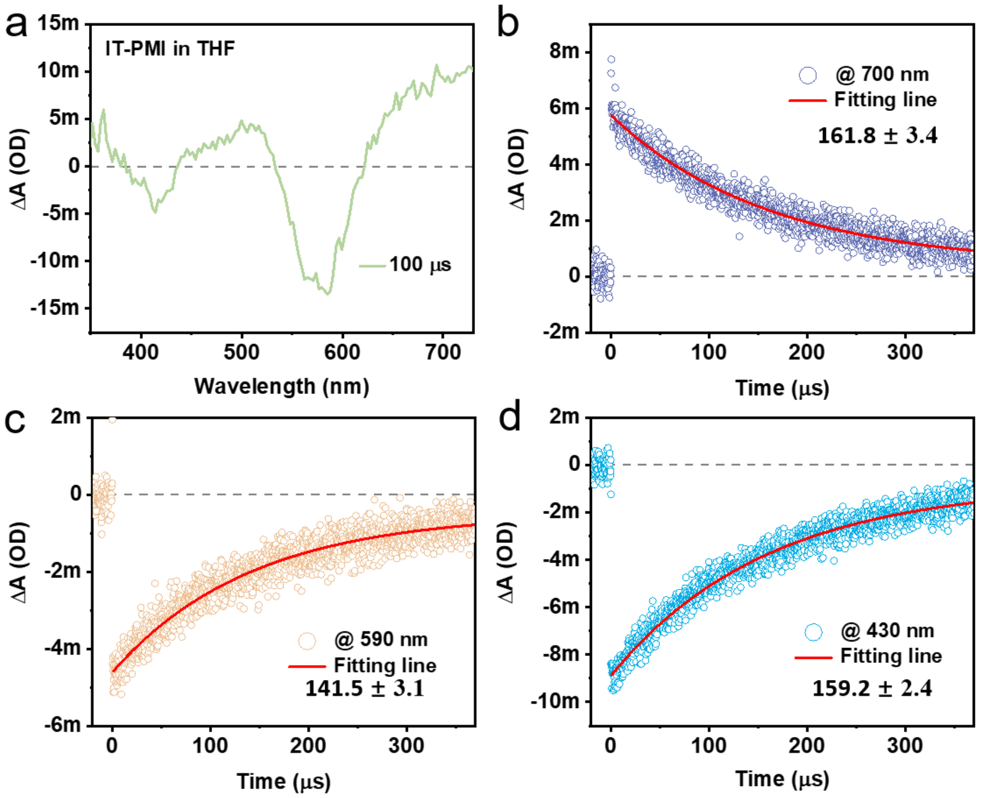

Figure S27. ns-TA spectra and selected kinetics of IT-PMI in THF, excitation at 550 nm (7 mJ pulse^{-1}).

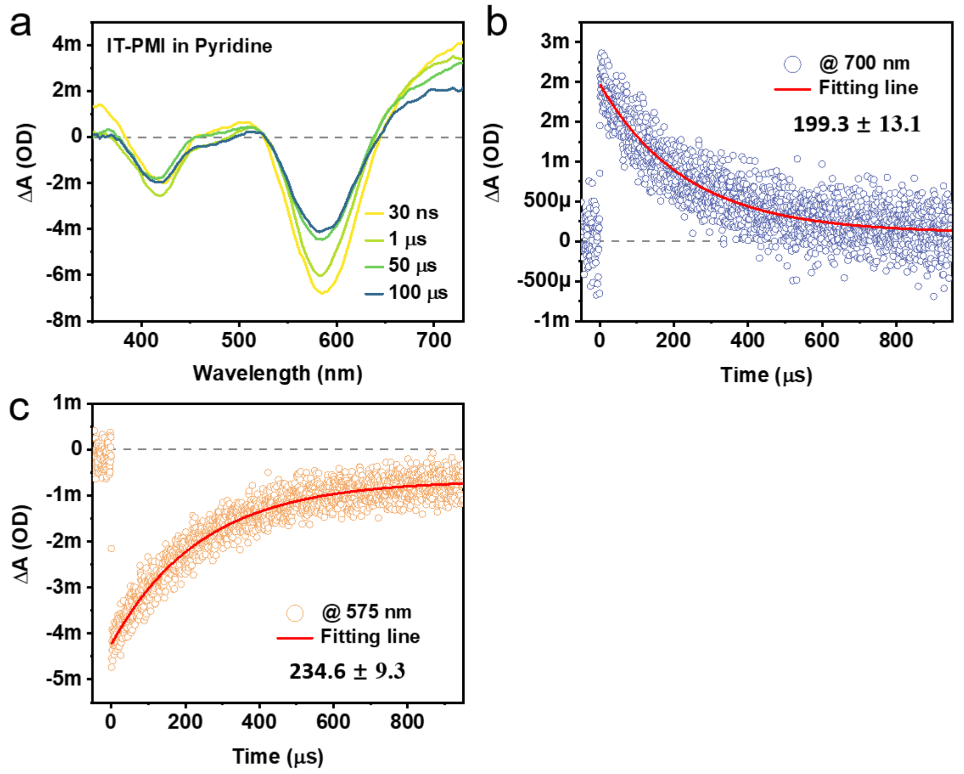

Figure S28. ns-TA spectra and selected kinetics of IT-PMI in pyridine, excitation at 530 nm (7 mJ pulse^{-1}).

10. EPR

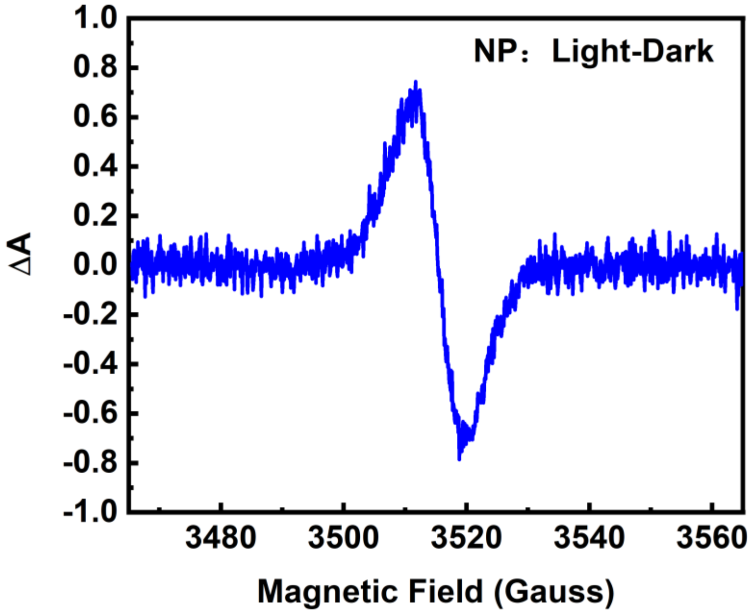

Figure S29. Continuous-wave electron paramagnetic resonance (CW-EPR) measurements were performed on freeze-dried IT-PMI nanoparticle powders. The EPR signal was recorded under argon atmosphere, with the light-induced signal obtained by subtracting the dark signal from the illuminated condition.

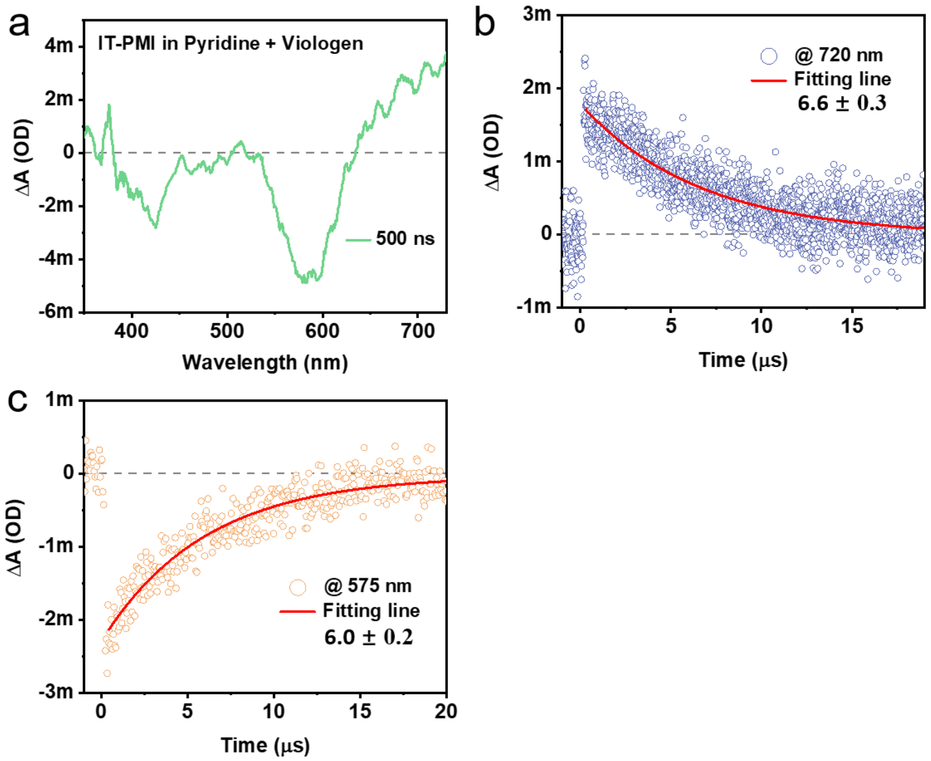

Figure S30. ns-TA spectra and selected kinetics of IT-PMI with adding methyl viologen as electron acceptor in pyridine, excitation at 530 nm (7 mJ pulse^{-1}).

11. Electrochemical and Spectro-electrochemistry tests

11.1 Photoinduced charge transfer driving force

Electrochemical measurements in various solvents were performed to evaluate the thermodynamic driving forces for the CS state, based on the Weller equation. Specifically, cyclic voltammetry (CV) or differential pulse voltammetry (DPV) was carried out for IT-PMI in high-polarity solvent pyridine, medium-polarity solvent THF, and in the solid state (as a film deposited on FTO). Due to the lack of a suitable supporting electrolyte, direct electrochemical measurement in low-polarity solvent toluene was challenging; Instead, the redox potential was estimated by applying a solvation correction to the THF data.

To estimate the donor–acceptor distance, we assumed homogeneous dispersion of IT-PMI in the organic solvents at a concentration of 0.2 mM, yielding an average separation of 19.4 nm. This concentration is justified by the high extinction coefficient of IT-PMI (above 1×10^4 across most of its UV–vis absorption spectrum), which ensures ~99% light absorption according to the Beer–Lambert law. Solvation correction was only applied for toluene, as the same solvent environment was used in THF, pyridine, and the solid-state samples for both electrochemical and optical measurements. The CS state energies derived from the Weller equation:

$$\Delta G_{ET} = E_{Ox} - E_{Red} - E_{0-0} - C + S$$

$$C = \frac{e^2}{4\pi r_{D-A}\varepsilon_0\varepsilon_S}$$

$$S = \frac{e^2}{8\pi\varepsilon_0}\left(\frac{1}{r_D} + \frac{1}{r_A}\right)\left(\frac{1}{\varepsilon_S} - \frac{1}{\varepsilon_P}\right)$$

Wherein, ΔG_{ET} is the Gibbs energy of photoinduced electron-transfer driving force; C is the Coulomb interaction correction term; S is the solvation correction term; r_{D-A} is the distance from the donor center to acceptor center (10.8 Å), estimated from the single crystal packing; $\varepsilon_0 \approx 8.854 \times 10^{-12} C^2 J^{-1} m^{-1}$ is the vacuum permittivity; ε_S or $\varepsilon_P \approx 3, 2.38, 8.93, 12.4$ are the relative permittivity of IT-PMI, Toluene, THF and Pyridine, respectively; e is the elementary charge; r_D and r_A is the ionic radii of donor and acceptor, respectively.

Table S6. Photoinduced charge transfer driving force in solvents under varied polarities, as well as aggregate state in NPs.

IT-PMI	E_{Ox}	E_{Red}	E_{0-0}	C	S	ΔG_{ET}
Toluene	*1.12 V*	*-0.73 V*	2.02 eV	0.03 eV	0.31 eV	0.11 eV
THF	1.12 V	-0.73 V	1.94 eV	0.008 eV	0 eV	-0.10 eV
Pyridine	1.08 V	-0.66 V	1.93 eV	0.006 eV	0 eV	-0.20 eV
Nanoparticle	1.32 V	-0.67 V	1.90 eV	0.45 eV	0 eV	-0.36 eV

11.2 Spectro-electrochemistry of IT-PMI measured in THF as the monomer state, and IT-PMI NPs measured in water as the nanoparticle state

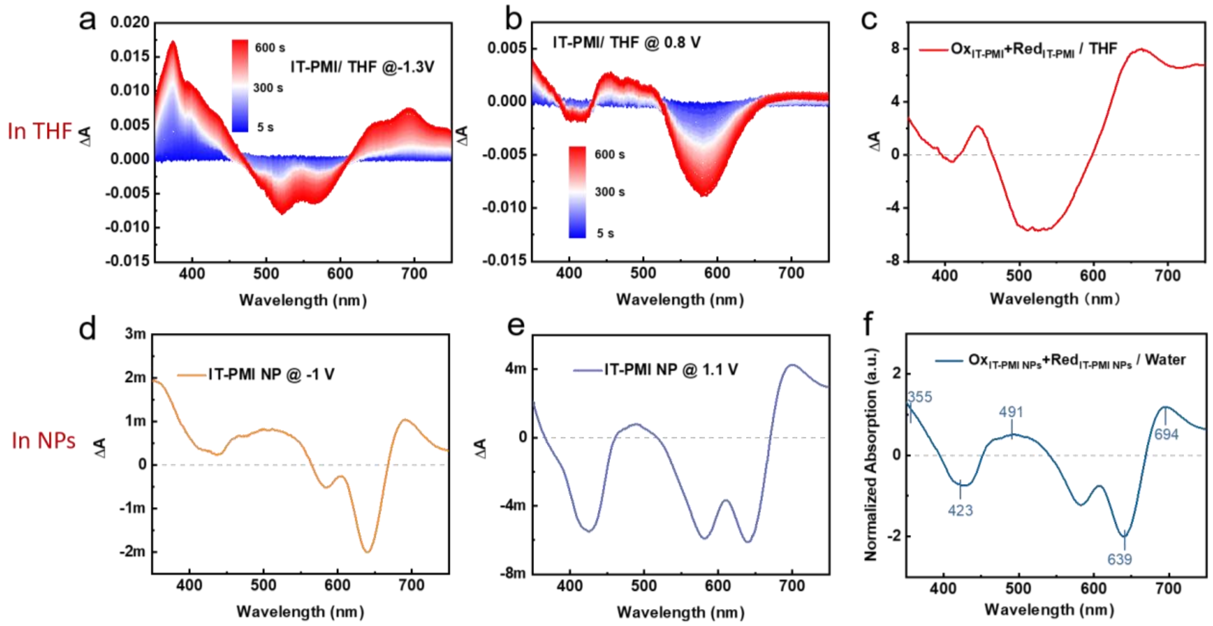

Figure S31. Spectro-electrochemistry (a) and (d) Reduced species absorption; (b) and (e) Oxidized species absorption; (c) and (f) Oxidized species and reduced species absorption of IT-PMI measured in THF as the monomer state and IT-PMI NPs measured in water as the nanoparticle state, respectively.

Spectroelectrochemistry of the IT-PMI measured in THF, with a bias of -1.3 V vs. Fc^{+}/Fc for reduced species and a bias of 0.8 V vs. Fc^{+}/Fc for oxidized species, with Pt mesh as the working electrode, Pt wire as the counter electrode, $Ag/AgNO_3$ as the reference electrode, 0.1 M TBAPF6 as the supporting electrolyte under Ar condition.

Spectroelectrochemistry of the IT-PMI NPs in water, with a bias of -1V vs. Fc^{+}/Fc for reduced species and a bias of 1.1 V vs. Fc^{+}/Fc for oxidized species, with Pt mesh as the working electrode, Pt wire as the counter electrode, Ag/AgCl as the reference electrode, 0.1 M KCl as the supporting electrolyte under Ar condition.

11.3 Electrochemistry of the IT-PMI in solution and IT-PMI film in in acetonitrile

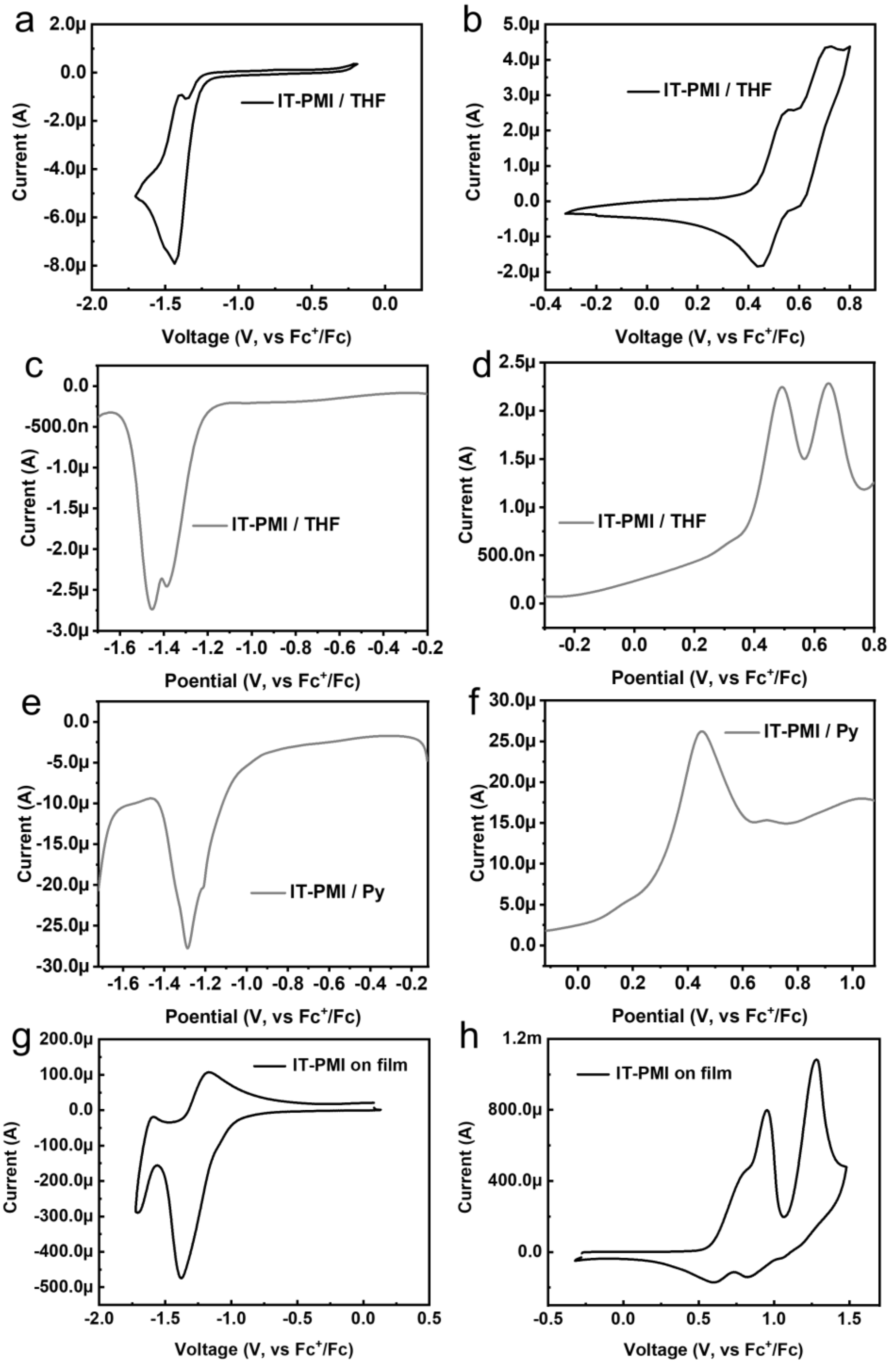

Figure S32. Cyclic voltammetry (a), (b) and Differential potential voltammetry (c), (d) of the IT-PMI measured in THF; Differential potential voltammetry (e), (f) of the IT-PMI measured in PV; Cyclic voltammetry of IT-PMI film on FTO measured in ACN (g), (h), with 0.2 M $TBAPF_6$ as the supporting electrolyte and $Ag/AgNO_3$ as the reference electrode, and glassy carbon as the working electrode. CV with a scan rate of 50 mV s^{-1}, and DPV with a scan rate of 5 mV s^{-1}, Ferrocene as the external reference.

Table S7. Photophysical parameters of IT-PMI.

	λ_{max}/nm (ε, M^{-1} cm^{-1})	E_{0-0} (eV)	E_{S/S^-} (V vs. NHE)	E_{S^*/S^-} (V vs. NHE)	$E_{S^+/S}$ (V vs. NHE)	E_{S^+/S^*} (V vs. NHE)
**Solution**	506 (8.96×10^4)	2.0	-0.73	1.27	1.12	-0.88
**Film**		1.9	-0.67	1.23	1.32	-0.58

All redox potentials are relative to the NHE, with a formula as follows:

$$E\ (\text{vs NHE}) = E\ (\text{vs Ag/AgNO}_3) - E\ (\text{Fc vs Ag/AgNO}_3) + E\ (\text{Fc vs NHE})$$

Here, in the right equation, the first part is obtained from the electrochemistry measurement in THF, the second part is the ferrocene potential as the external standard under the same conditions, the third part E (*Fc vs* NHE) = 0.63 V when measured in THF, which is obtained from the reference.

$$E_{S^*/S^-} = E_{S/S^-} + E_{0-0}$$

$$E_{S^+/S^*} = E_{S^+/S} - E_{0-0}$$

13. $^{1}O_2$ probing experiments

13.1 Absorption with ABDA probe

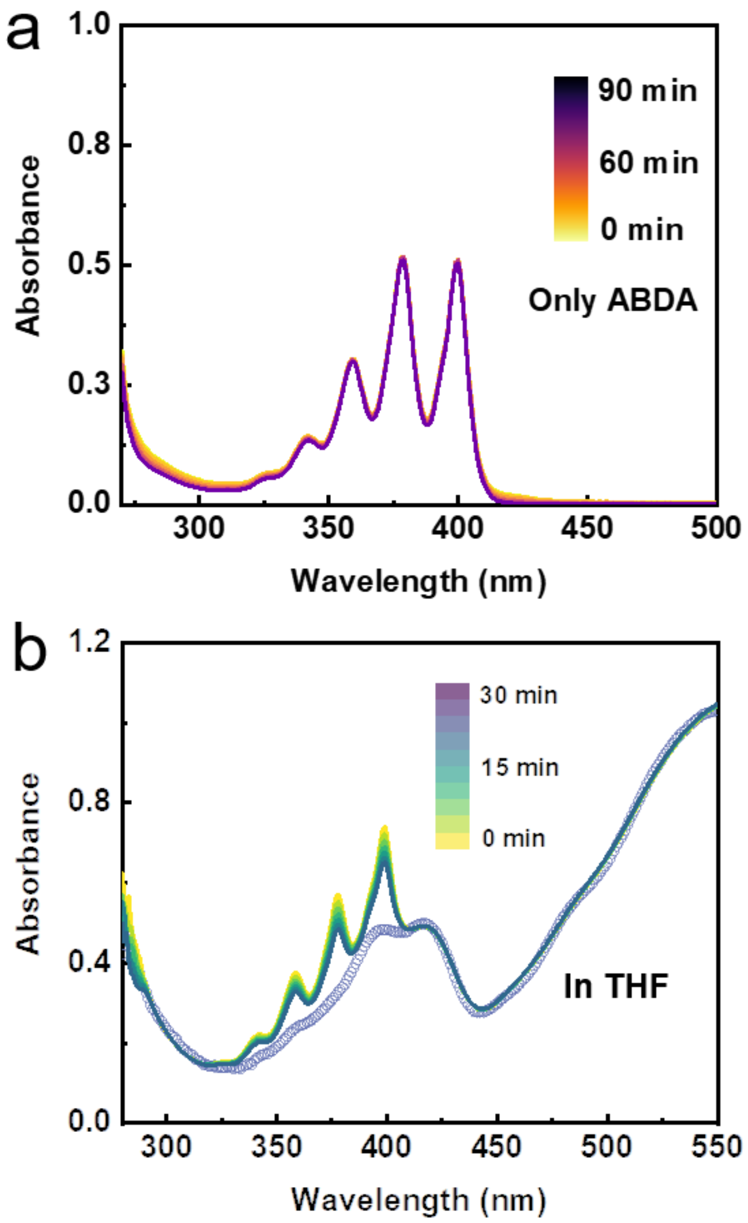

Figure S33. UV-vis absorbance evolution under continues LED light illumination (a) Pure 9,10-anthracenediyl-bis(methylene)dimalonic acid (ABDA) in water after purging O_2; (b) ABDA with IT-PMI in THF after purging O_2, the blue circle represents pure IT-PMI absorption in THF.

13.2 $^1O_2$ PL emission test

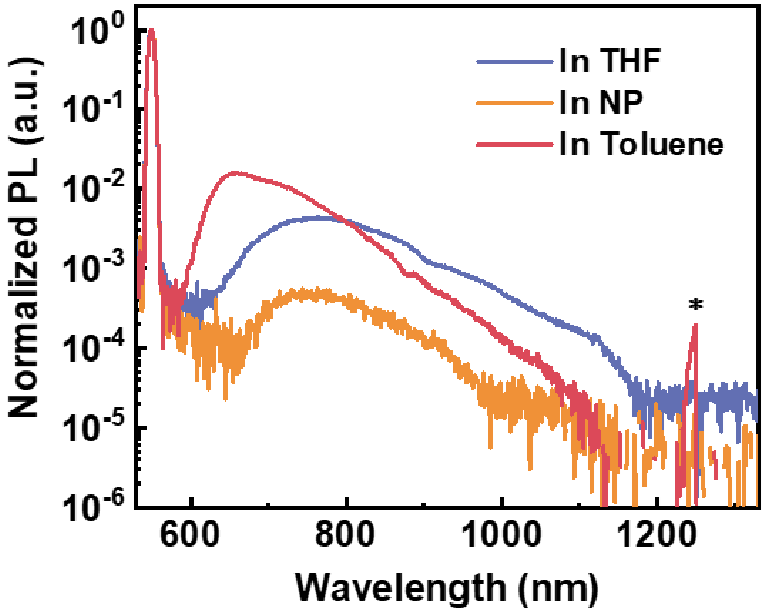

Figure S34. $^1O_2$ PL emission measurements of IT-PMI in Toluene, THF, and IT-PMI NPs in water, under excitation at 550 nm with sample absorbance of 0.15, all measurements are performed in FLS1000 with an integrating sphere, the broad PL band in the middle attributed to the PL emission from IT-PMI under different conditions, the peak at 1265 nm is the $^1O_2$ PL emission, and only found in toluene sample.

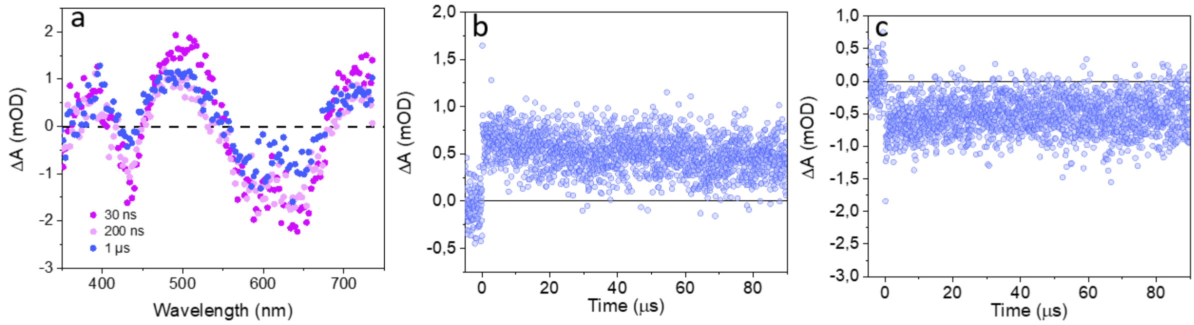

Figure S35. ns-TA results for IT-PMI NPs in water saturated with O_2 upon 530 nm excitation (10 mJ pulse^{-1}). (a) Spectral evolution at selected time delays (b) Kinetic trace monitored at 500 nm. (c) Kinetic trace monitored at 645 nm.

14. fs-TA profile comparison of IT-PMI NPs

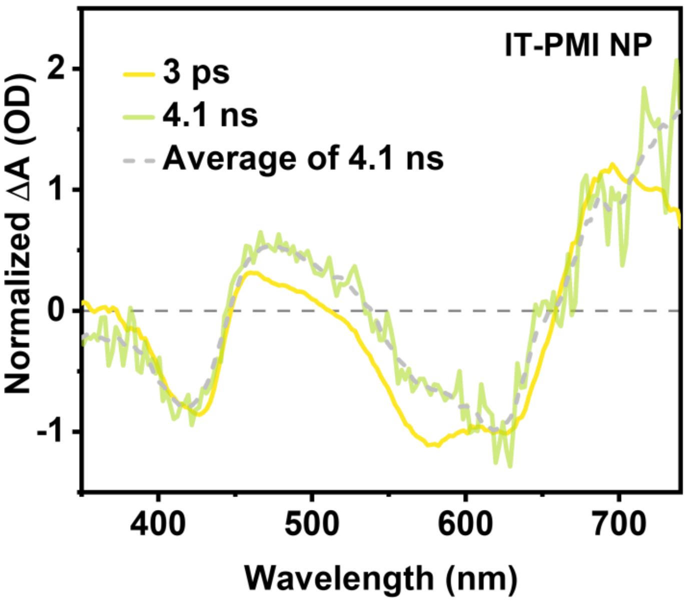

Figure S36. Absorption profile comparison of IT-PMI NP obtained in fs-TA under two distinguish time scales, as profile at 3 ps represents ICT state (yellow line), and profile at 4.1 ns represent CS state (green line), the dot line is averaged smooth profile at 4.1 ns. Distinguish differences could be found, and profile at 4.1 ns more closely resemble to profile from SEC.

15. Examples for the reported charge separated state lifetime for nano-assemblies

Table S8. Examples of the reported charge separated state lifetime in nano-assemblies

System	Donor	Acceptor	CS state lifetime	Reference
Π-stacked self-assembly	Porphyrin-perylenediimide A_4-D		169 ns	*Angew. Chem. Int. Ed.*, 2014, 53, 3457[9]
Non-covalent assembly (two components)	TPPS	C_{60}	3.16 µs	*Adv. Mater.*, 2021 ,33, 31, 2101026[10]
Nanoribbon	Carbazole-naphthalimide dyad		16 ns	*Angew. Chem. Int. Ed.*, 2022, 61, 12, e202117645[11]
Donor/acceptor Nanoparticle (two components)	PM6 (polymer)	Y6	second scale	*Nature Energy,* 2022, 7, 340[12]
Non-covalent assembly (two components)	Zinc porphyrin	Fullerene	220.9 ps	*Applied Catalysis B: Environmental,* 2023, 324, 122284[13]
Donor/acceptor Nanoparticle (two components)	PFODTBT (polymer)	ITIC	431 ps	*Phys. Chem. Chem. Phys.*, 2023,25, 2935[14]
Dyad Nanoparticle	Porphyrin-viologen dyad		4.3 ms	*J. Am. Chem. Soc.*, 2023, 145, 34, 18687[15]
2D Perovskite Nanoplatelet (two components)	Phenothiazine	Perovskite	68 ns	*J. Phys. Chem. Lett.*, 2023, 14, 9, 2241[16]
Nanocrystal	Perylene diimide dimer		19.6 ns	*J. Am. Chem. Soc., 2025, 147, 15, 12730*[17]
Nanocrystal	PMI based A-D-A molecule		1.2 s	***This work***

16 Photocatalytic H_2 evolution

16.1. Standard calibration curve for H_2 amount

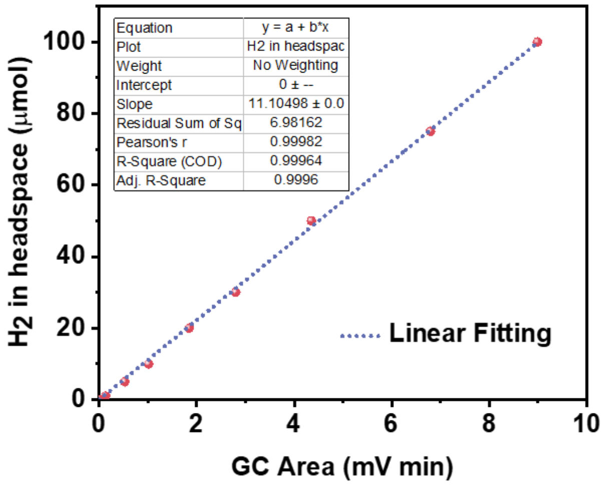

Figure S37. H_2 calibration curve obtained from injecting certain amount of Ar diluted H_2 into GC and calculating the integration area though Thermal Conductivity Detector.

16.2 HER on varied concentration of AA, Pt, and IT-PMI NPs

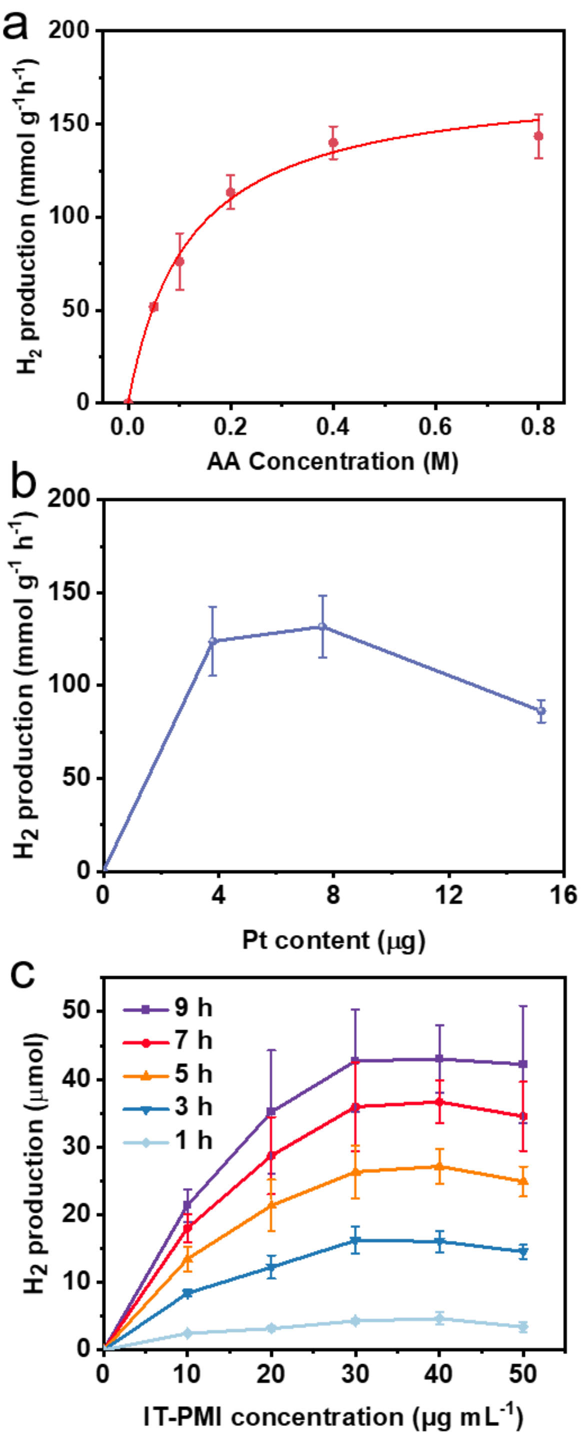

Figure S38. Photocatalytic H_2 production of IT-PMI NPs under LED light (420 – 750 nm, 50 mW cm^{-2}), pH=4, (a) Ascorbic acid concentration dependent photocatalytic H_2 production, with the maximum HER rate at 0.8 M of 143.5±11.6 mmol g^{-1} h^{-1}; (b) Pt content dependent photocatalysis H_2 production, data averaged under 5 hours, with the maximum HER rate at 7.6 μg Pt of 131.7±16.6 mmol g^{-1} h^{-1}; (c) IT-PMI nanoparticle concentration dependent

photocatalysis H_2 production, with the total average HER rate of 342.7±25.2 mmol g^{-1} h^{-1}, all data averaged from 4 independent batches.

16.3 Cryo-EM images of IT-PMI NPs with Pt as co-catalyst

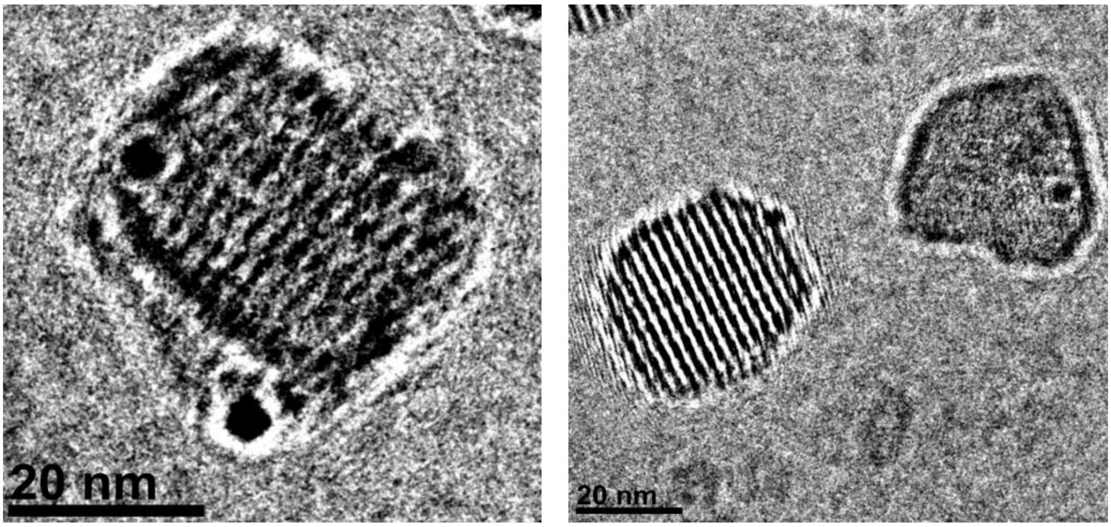

Figure S39. Cryo-EM images of IT-PMI NPs deposited Pt by photolysis, most Pt nanoparticles are deposited on the edge of the IT-PMI NPs.

16.4 Concentration-dependent light harvesting efficiency

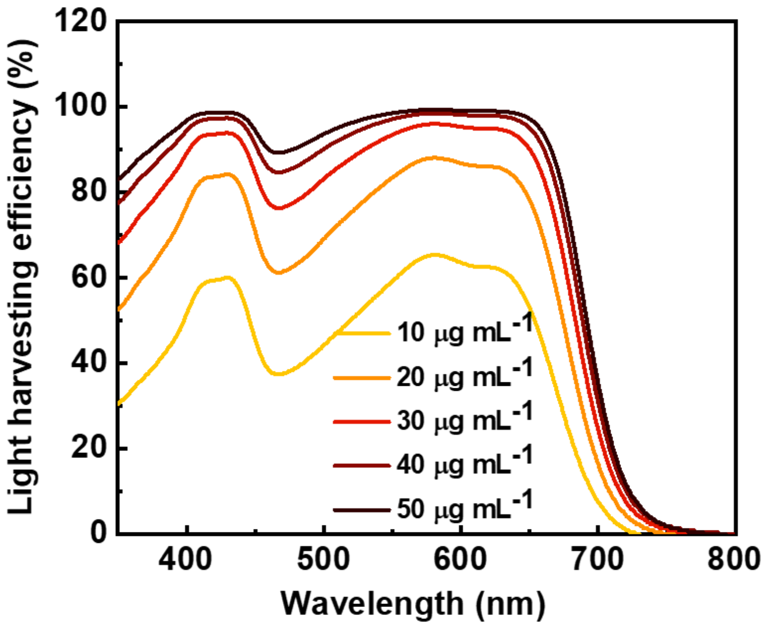

Figure S40. Light harvesting efficiency of various concentration IT-PMI dots, 1.5 mL stock solution with the noted concentration added with 0.5 mL water, optical path length is 2 cm, similar to photocatalytic vial.

17. ns-TA quenching experiments with ascorbic acid (pH 4) and Pt

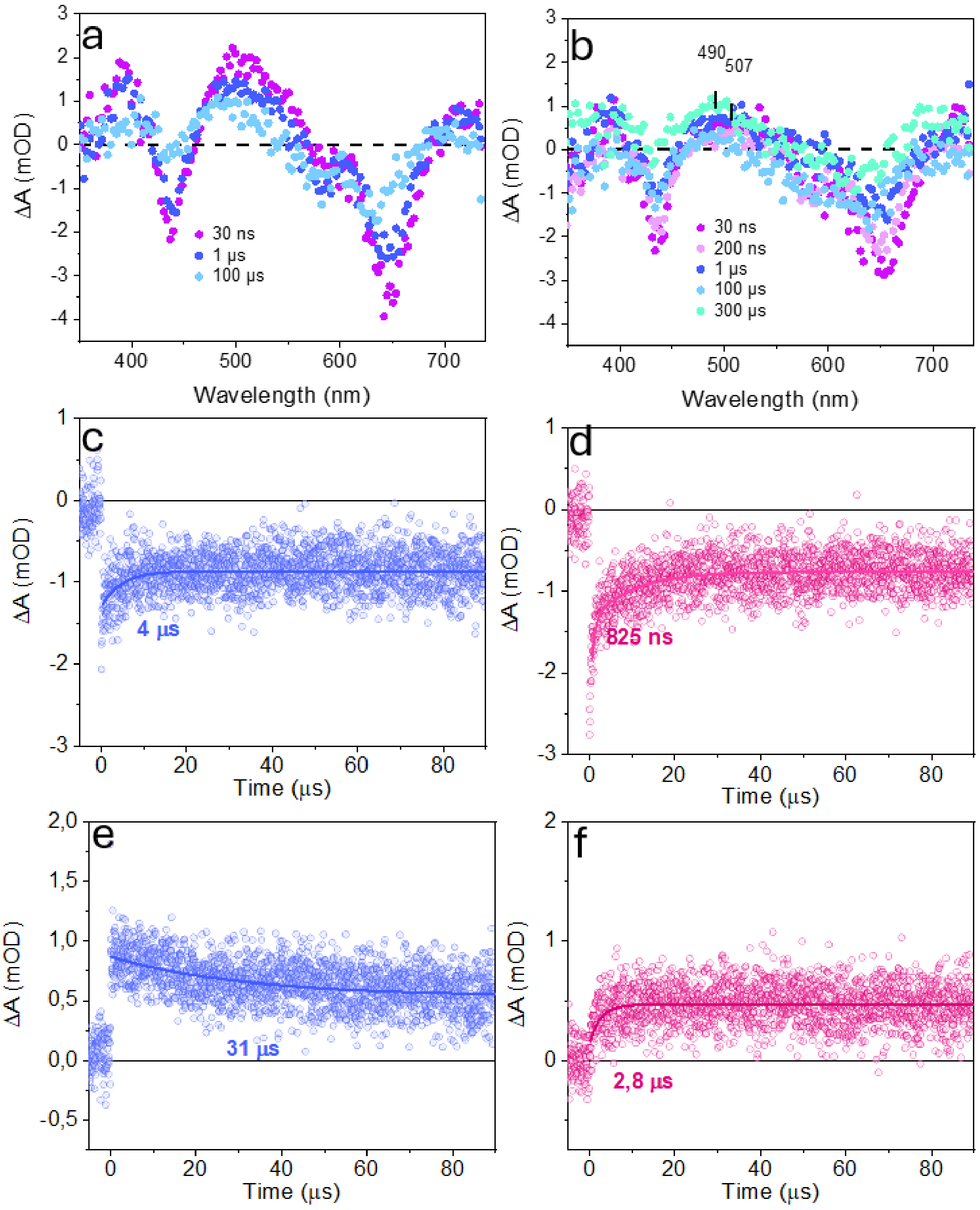

Figure S41. ns-TA spectra of IT-PMI NPs in water after Ar purging upon 530 nm excitation (10 mJ pulse^{-1}; absorbance at the excitation wavelength was kept similar across samples): (a) without ascorbic acid (AA); (b) with AA; (c) kinetic trace at 645 nm without AA; (d) kinetic trace at 645 nm with AA; (e) kinetic trace at 500 nm without AA; and (f) kinetic trace at 500 nm with AA.

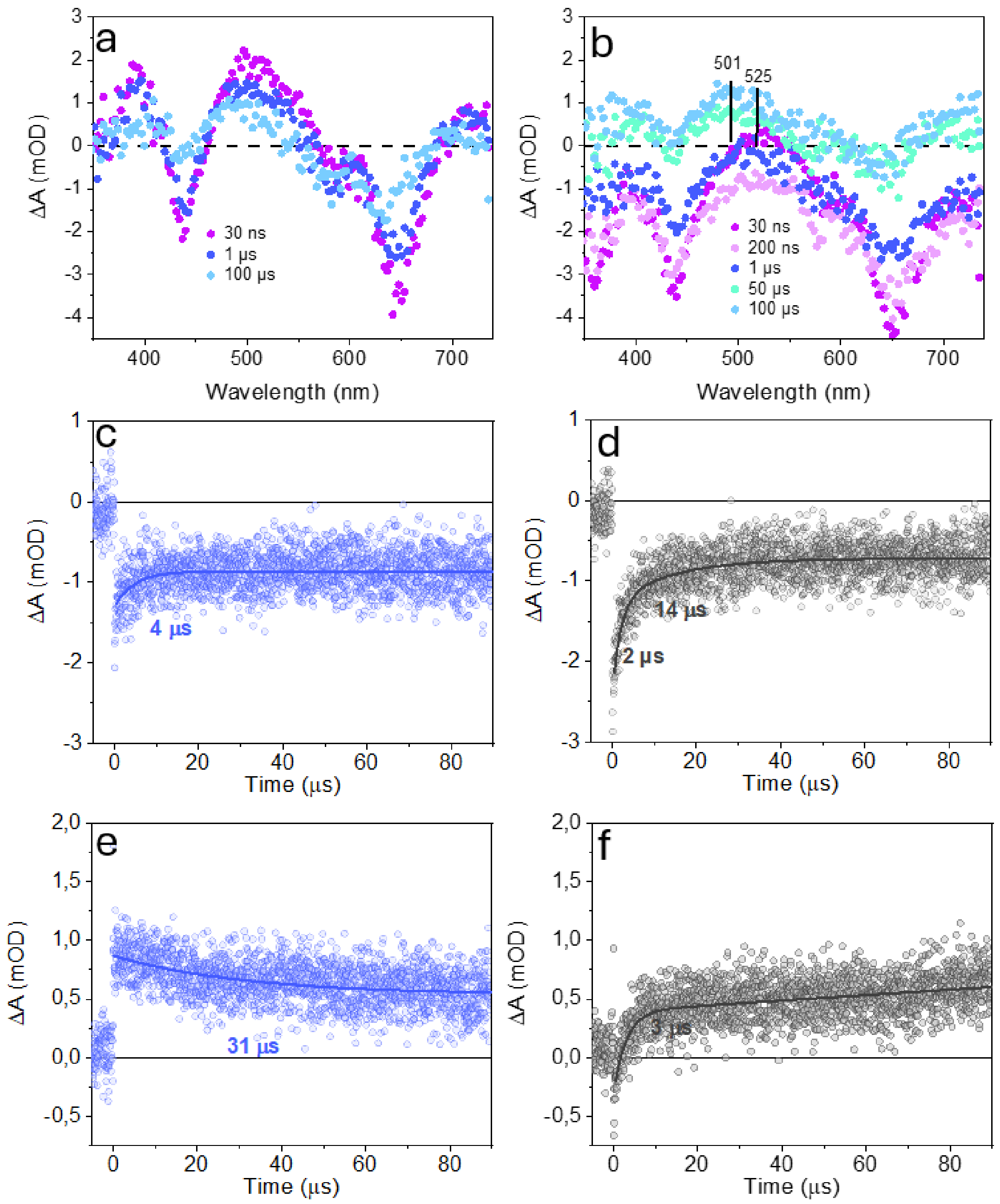

Figure S42. ns-TA spectra of IT-PMI NPs in water after Ar purging upon 530 nm excitation (10 mJ pulse^{-1}; absorbance at the excitation wavelength was kept similar across samples): (a) without photodeposited Pt; (b) with photodeposited Pt; (c) kinetic trace at 645 nm without photodeposited Pt; (d) kinetic trace at 645 nm with photodeposited Pt; (e) kinetic trace at 500 nm without photodeposited Pt; and (f) kinetic trace at 500 nm with photodeposited Pt.

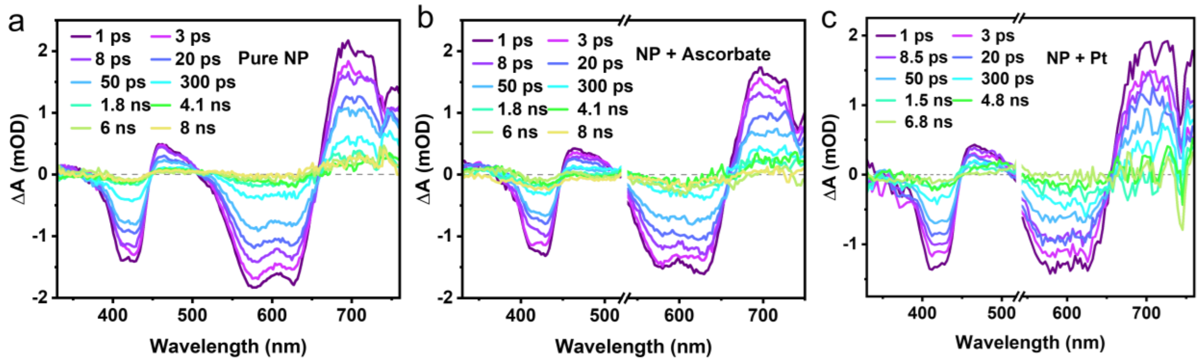

Figure S43. fs-TA spectra of IT-PMI NPs in water after Ar purging upon 530 nm excitation (19.7 nJ/pulse, absorbance at the excitation wavelength were kept similar across samples): (a) Pure IT-PMI NP in water; (b) with AA; (c) with Pt (with some scattering due to Pt aggregates).

18. Stability tests

18.1 Light stability test of nanoparticles

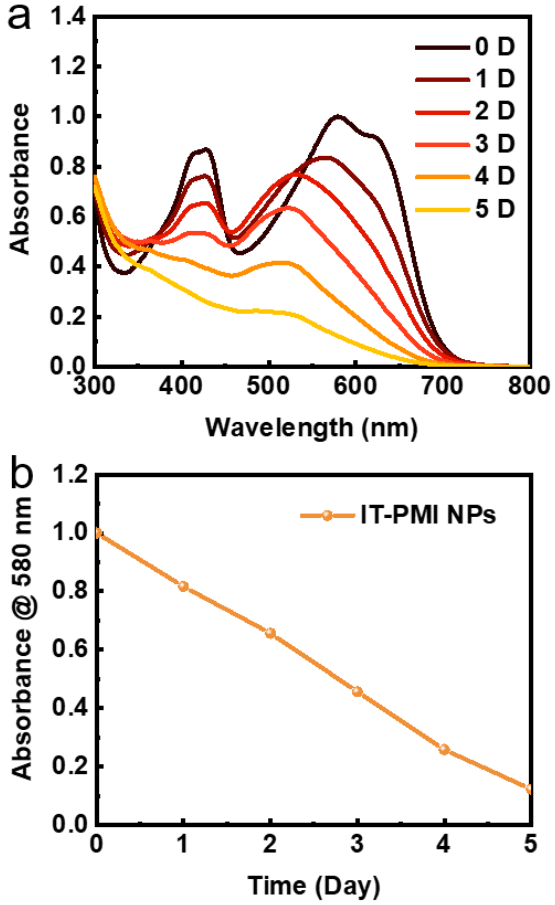

Figure S 44. (a) UV-vis absorbance evolution of the IT-PMI NPs under light illumination in the absence of AA and Pt; (b) UV-vis absorbance change traced at 580 nm of IT-PMI NPs dispersed in water in the absence of AA and Pt, illuminated under continues LED light.

18.2 Light stability test of nanoparticles

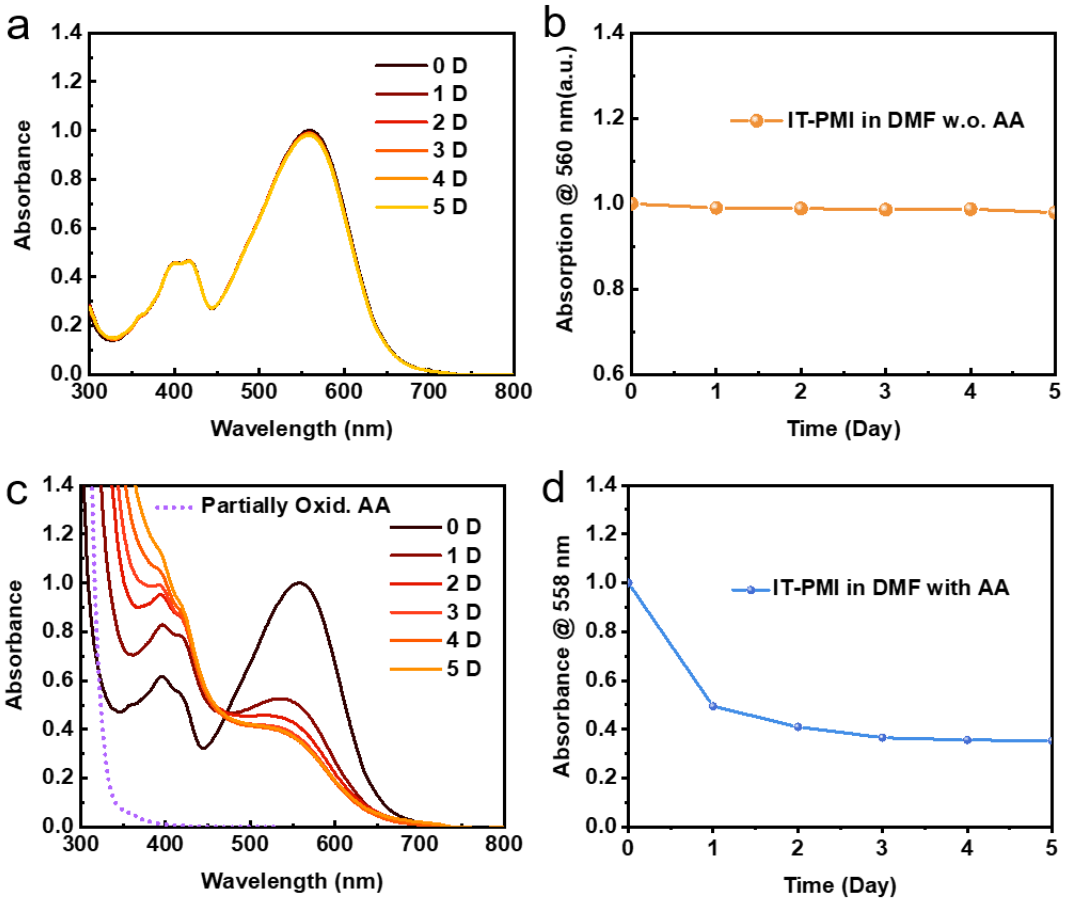

Figure S 45. UV-vis absorbance evolution of the IT-PMI in DMF solution as monomer after Ar purging under light illumination (a) without adding AA; (b) with adding AA; UV-vis absorbance change traced at 558 nm of IT-PMI in DMF solution as monomer after Ar purging, illuminated under continues LED light. (c) without adding AA; (b) with adding AA.

19. Setup of scalable HER using IT-PMI NPs

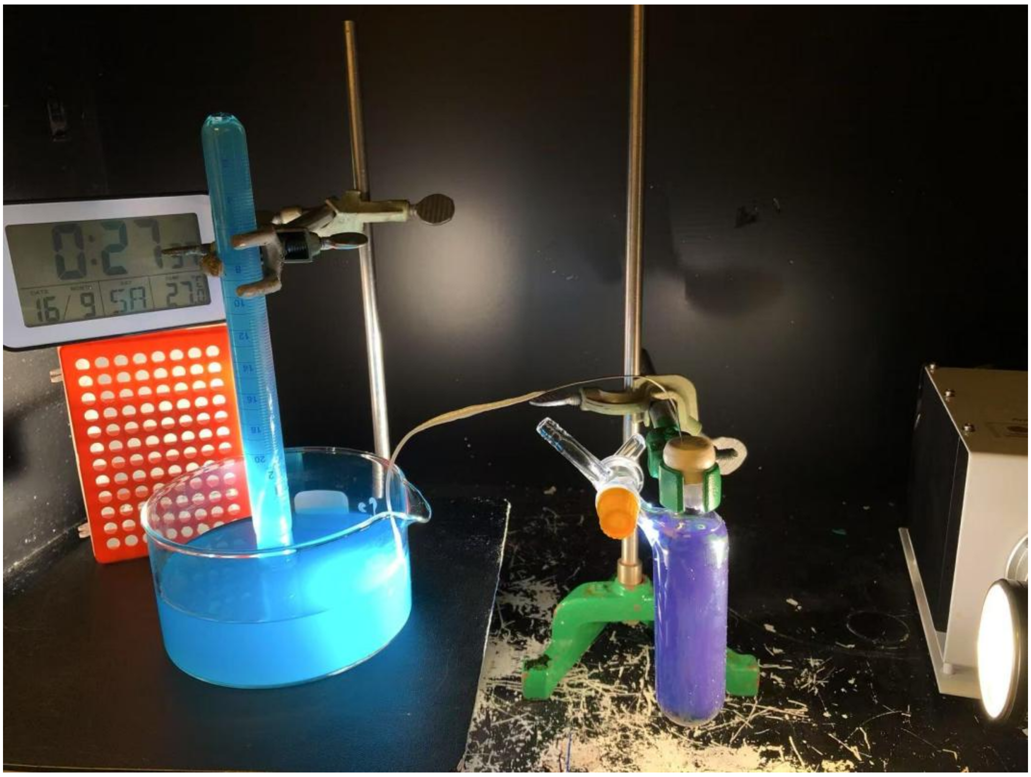

Figure S 46. Photocatalytic HER with IT-PMI NPs using a scalable setup, the produced H2 was collected with a cylinder by water displacement method.

20. External quantum efficiency (EQE) measurement

The external quantum efficiency of H_2 production photocatalysis was performed under a Xenon light with different mono-light filters, and the light intensity was calibrated with a power meter, the EQE was calculated based on the following equation:

$$\mathrm{EQE} = \frac{\mathrm{N_{electron}}}{\mathrm{N_{photon}}} * 100\%$$

$$\mathrm{N_{electron}} = 2 * \mathrm{n_{H_2}(mol)} * \mathrm{N_A(mol^{-1})}$$

$$\mathrm{N_{photon}} = \frac{\mathrm{P(W\,cm^{-2})} * \mathrm{A(cm^2)} * \mathrm{t(s)}}{{}^{1243}\!/_{\lambda(\mathrm{nm})} * 1.602 \times 10^{-19}(\mathrm{J})}$$

Where, $\mathrm{N_{electron}}$ is the number of generated electrons, $\mathrm{N_{photon}}$ is the number of the incident photons, $\mathrm{n_{H_2}}$ is the produced H_2 in molar, $\mathrm{N_A}$ is the Avogadro's number, P is the lamp power, A is the illuminated area of the sample, t is the illuminated time, λ is the incident light wavelength.

21. For TON and TOF calculation

For TON calculation:

$$\mathrm{TON} = \frac{\mathrm{n_{H_2}}}{\mathrm{n_{IT-PMI\,NP}}}$$

For TOF calculation:

$$\mathrm{TOF} = \frac{\mathrm{n_{H_2}}}{\mathrm{n_{IT-PMI\,NP}} * t}$$

The number of IT-PMI NPs was estimated based on $\mathrm{n_{IT-PMI\,NP}} = \frac{m_{total}}{\rho * V_{NP}}$, where m_{total} is the total mass of the IT-PMI NPs in the applied system, which is determined by the UV-vis absorption calibration curve; ρis the density of the nanoparticle, perylene crystal density value has been applied as they share similar structure [18,19], 1.3 g cm^{-3}; V_{NP} is the nanoparticle size, which determined by the average size from cryo-EM images (average diameter around 35 nm, dice structure as it determined).

Reference

1 Cai, B. *et al.* An Indacenodithieno[3,2-b]thiophene-based Organic Dye for P-type Dye-Sensitized Solar Cells and Photoelectrochemical H_2O_2 Production. *ChemPhotoChem* **8**, e202300297, doi:https://doi.org/10.1002/cptc.202300297 (2024).

2 Gaussian 16 Rev. C.01 (Wallingford, CT, 2016).

3 Yanai, T., Tew, D. P. & Handy, N. C. A new hybrid exchange–correlation functional using the Coulomb-attenuating method (CAM-B3LYP). *Chemical Physics Letters* **393**, 51-57, doi:https://doi.org/10.1016/j.cplett.2004.06.011 (2004).

4 Tomasi, J., Mennucci, B. & Cammi, R. Quantum Mechanical Continuum Solvation Models. *Chemical Reviews* **105**, 2999-3094, doi:10.1021/cr9904009 (2005).

5 Almgren, M., Edwards, K. & Karlsson, G. Cryo transmission electron microscopy of liposomes and related structures. *Colloids and Surfaces A: Physicochemical and Engineering Aspects* **174**, 3-21, doi:https://doi.org/10.1016/S0927-7757(00)00516-1 (2000).

6 Liu, A. *et al.* Panchromatic Ternary Polymer Dots Involving Sub-Picosecond Energy and Charge Transfer for Efficient and Stable Photocatalytic Hydrogen Evolution. *Journal of the American Chemical Society* **143**, 2875-2885, doi:10.1021/jacs.0c12654 (2021).

7 Förster, T. Zwischenmolekulare Energiewanderung und Fluoreszenz. *Annalen der Physik* **437**, 55-75, doi:https://doi.org/10.1002/andp.19484370105 (1948).

8 Lu, T. & Chen, F. Multiwfn: A multifunctional wavefunction analyzer. *Journal of Computational Chemistry* **33**, 580-592, doi:https://doi.org/10.1002/jcc.22885 (2012).

9 Roznyatovskiy, V. V., Carmieli, R., Dyar, S. M., Brown, K. E. & Wasielewski, M. R. Photodriven Charge Separation and Transport in Self-Assembled Zinc Tetrabenzotetraphenylporphyrin and Perylenediimide Charge Conduits. *Angewandte Chemie International Edition* **53**, 3457-3461, doi:https://doi.org/10.1002/anie.201309335 (2014).

10 Yang, J., Jing, J. & Zhu, Y. A Full-Spectrum Porphyrin–Fullerene D–A Supramolecular Photocatalyst with Giant Built-In Electric Field for Efficient Hydrogen Production. *Advanced Materials* **33**, 2101026, doi:https://doi.org/10.1002/adma.202101026 (2021).

11 Lin, H. *et al.* Molecular Dipole-Induced Photoredox Catalysis for Hydrogen Evolution over Self-Assembled Naphthalimide Nanoribbons. *Angewandte Chemie International Edition* **61**, e202117645, doi:https://doi.org/10.1002/anie.202117645 (2022).

12 Kosco, J. *et al.* Generation of long-lived charges in organic semiconductor heterojunction nanoparticles for efficient photocatalytic hydrogen evolution. *Nature Energy* **7**, 340-351, doi:10.1038/s41560-022-00990-2 (2022).

13 Jing, J., Li, J., Su, Y. & Zhu, Y. Non-covalently linked donor-acceptor interaction enhancing photocatalytic hydrogen evolution from porphyrin assembly. *Applied Catalysis B: Environmental* **324**, 122284, doi:https://doi.org/10.1016/j.apcatb.2022.122284 (2023).

14 Liu, A. *et al.* Excited-state and charge-carrier dynamics in binary conjugated polymer dots towards efficient photocatalytic hydrogen evolution. *Physical Chemistry Chemical Physics* **25**, 2935-2945, doi:10.1039/D2CP04204E (2023).

15 Cai, B. *et al.* Promoted Charge Separation and Long-Lived Charge-Separated State in Porphyrin-Viologen Dyad Nanoparticles. *Journal of the American Chemical Society* **145**, 18687-18692, doi:10.1021/jacs.3c04372 (2023).

16 He, S., Jin, T., Ni, A. & Lian, T. Electron Trapping Prolongs the Lifetime of Charge-Separated States in 2D Perovskite Nanoplatelet-Hole Acceptor Complexes. *The Journal of Physical Chemistry Letters* **14**, 2241-2250, doi:10.1021/acs.jpclett.2c03815 (2023).

17 Mao, J. *et al.* Ultrasmall Organic Nanocrystal Photocatalyst Realizing Highly Efficient Symmetry Breaking Charge Separation and Transport. *Journal of the American Chemical Society* **147**, 12730-12739, doi:10.1021/jacs.5c01205 (2025).

18 Donaldson, D. M., Robertson, J. M. & White, J. The crystal and molecular structure of perylene. *Proceedings of the Royal Society of London. Series A. Mathematical and Physical Sciences* **220**, 311-321, doi:10.1098/rspa.1953.0189 (1997).

19 Matsumoto, A. *et al.* Aromaticity Relocation in Perylene Derivatives upon Two-Electron Oxidation To Form Anthracene and Phenanthrene. *Chemistry – A European Journal* **22**, 14462-14466, doi:https://doi.org/10.1002/chem.201602188 (2016).
